# The effectiveness of implementation intentions in children: A systematic review and meta‐analysis

**DOI:** 10.1111/bjop.70065

**Published:** 2026-03-05

**Authors:** Jasmin Breitwieser, Tilman Reinelt

**Affiliations:** ^1^ Child Development in Educational Contexts, DIPF Leibniz Institute for Research and Information in Education Frankfurt am Main Germany; ^2^ Center for Individual Development and Adaptive Education of Children at Risk (IDeA) Frankfurt am Main Germany; ^3^ Department of Neonatology University of Zurich Zurich Switzerland

**Keywords:** children, if–then plans, implementation intentions, meta‐analysis

## Abstract

Self‐regulation abilities in childhood are important for long‐term academic achievement and healthy development. As self‐regulatory abilities are still developing, ‘simple’ interventions are needed to foster self‐regulation. Implementation intentions are simple plans that could promote goal achievement in children. However, little is known about their overall effectiveness and moderating factors in children. This registered report presents a systematic review of the literature and a meta‐analysis of the effects of implementation intentions on children's goal achievement. The meta‐analysis included a total of 52 effect sizes from 42 studies (*N* = 12,957, *M*_age_ = 10.67 years), published between 1975 and 2025. Overall, we found a small‐to‐medium effect of implementation intentions in children (Hedge's *g* = 0.31, 95% CI [0.21; 0.41]). Study heterogeneity was high (*Q*(51) = 146.47, *p* < .001, *I* ^2^ = 65.2%). The effects were stronger in studies with younger children and (in some analyses) children with ADHD, suggesting that implementation intentions are particularly effective when self‐regulation abilities are limited. Future research should increase conceptual and methodological rigour. In addition, research and practice should aim to support children in forming strong mental representations of plans, generating high‐quality plans themselves and using implementation intentions as a meta‐strategy.

## BACKGROUND

From a very young age, children begin to think about the future. Preschoolers are already able to mentally represent future goals and formulate plans on how to pursue them (Atance & Metcalf, 2013; Haith, 1998). As children get older, their goals can become more long term and thus motivate future action (Dweck, 1986; Latham & Locke, 1979; Moersdorf et al., 2022). However, they are also more likely to compete with immediate gratification. Wishing to perform well at a test, for instance, requires repeated efforts and the prioritization of study time over more pleasurable activities. Resisting temptations, overcoming obstacles or inhibiting impulses requires self‐regulation. Children who are better at self‐regulation in early childhood are more likely to excel academically and socially, leading healthier lives with fewer behavioural problems (Duckworth et al., 2019; Moffitt et al., 2011; Perry et al., 2018). Thus, the development of self‐regulation in early childhood may pave the way for success later in life.

During early childhood, the development of self‐regulation is closely linked to brain development (Diamond, 2002; McClelland et al., 2018). As the frontal lobe and associated cognitive abilities continue to develop into late adolescence (Fuster, 2002), children have a harder time focusing their attention on goal‐relevant cues, shifting flexibly between different goals and inhibiting competing impulses (Diamond, 2013; Fuster, 2002; Luna et al., 2004). School entry also has an impact on the development of self‐regulation, with research suggesting that schooling can improve executive functions (McCrea et al., 1999; see also Morrison et al., 2010). Adolescence marks another sensitive period of self‐regulation development when adult‐like self‐regulatory strategies emerge (McClelland et al., 2018). At the same time, risk‐taking behaviour increases due to faster development of the socio‐emotional system compared to cognitive control (Steinberg, 2008). In addition, identity development and schooling lead to a shift in goal focus towards more long‐term and outcome‐related goals (Moersdorf et al., 2022; Wigfield et al., 2011). Taken together, the importance of self‐regulation for long‐term development and its variability in children (Montroy et al., 2016) make self‐regulation a main target for psychological interventions.

### Implementation intentions and successful goal striving

As cognitive functions develop during childhood and adolescence, self‐regulation interventions need to be simple and easy to use. Implementation intentions have been introduced as a ‘simple’ strategy that helps people translate their good intentions into action (Gollwitzer, 1999). It is grounded in action phase models that describe the motivational and volitional processes leading from intention formation to goal attainment (Gollwitzer, 1990; Heckhausen & Kuhl, 1985). Since intentions explain less than 30% of the variance in behaviour across behavioural domains (Sheeran, 2002), implementation intentions have been proposed as a volitional strategy that can help bridge this gap.

Implementation intentions are plans that link a situational cue to a goal‐directed action, typically as a statement of the form ‘If/when situation X occurs, (then) I will perform behavior Y’. For instance, the goal ‘I want to do well on the test next week!’ could be supplemented by an implementation intention that defines when and where to study for the test: ‘When I sit at my desk after school, I will open my textbook and start learning!’ Implementation intentions overlap with the concepts of action plans and coping plans, with action plans specifying when and how to act, and coping plans specifying strategies for overcoming obstacles (e.g. ‘If I feel bored by math, then I will remember its importance for the test’). While some researchers view action and coping plans as types of implementation intentions and often use these terms interchangeably (Carraro & Gaudreau, 2013; Sniehotta, 2009; Thompson et al., 2015), others note differences: While implementation intentions focus on specific cue–action links, action plans target more complex behavioural sequences and include instructions on how to perform them (Hagger & Luszczynska, 2014; Rhodes et al., 2020). For the purposes of this meta‐analysis, the essential aspect is that the intervention involves a plan connecting a specific cue to a goal‐directed action. These plans can address both internal (thoughts, feelings) and external (events, actions) cues and responses (see Bieleke et al., 2021).

Findings from laboratory experiments suggest two cognitive processes that mediate the effects of implementation intentions (for an overview, see Gollwitzer, 2014). First, implementation intentions have been shown to activate the mental representation of the situational cue specified in the if‐part of the plan, thus enhancing attention, memory and processing of the situational cue (e.g. Achtziger et al., 2012; Janczyk et al., 2015; Parks‐Stamm et al., 2007). Second, they have been shown to create a strong associative link between the cue and the action specified in the then‐part of the plan. Due to this link, the action is executed immediately, efficiently, independent of conscious intent and with reduced controllability (Bayer et al., 2009; Brandstätter et al., 2001; Gollwitzer & Brandstätter, 1997; Wieber & Sassenberg, 2006). Together, both mechanisms—increased cue accessibility and action automatization—explain the effectiveness of implementation intentions in promoting goal achievement.

### Are implementation intentions effective in children?

Meta‐analyses across various goal domains and demographics have consistently shown a positive effect of implementation intentions on goal achievement (Adriaanse et al., 2011; Bélanger‐Gravel et al., 2013; Chen et al., 2015; Gollwitzer & Sheeran, 2006; Malaguti et al., 2020; McWilliams et al., 2019; Silva et al., 2018; Toli et al., 2016). A recent meta‐analysis of meta‐analyses revealed a combined effect size of *d* = 0.54, 95% CI [0.51, 0.57], range: 0.24–0.99 (Keller et al., 2020).

However, the meta‐analyses performed to date are based almost exclusively on adult populations. One reason could be that some meta‐analyses focused on behaviours that are not relevant to the majority of children, such as smoking (McWilliams et al., 2019) or substance use (Malaguti et al., 2020). While other meta‐analyses investigated universally relevant goals such as emotion regulation (Webb et al., 2012) or healthy eating (Adriaanse et al., 2011), they incorporated almost no studies with children. The highest number of studies with children is found in a meta‐analysis focusing on individuals with mental health problems (Toli et al., 2016). However, while implementation intentions may be effective in children with attention‐deficit/hyperactivity disorder (ADHD), not much can be said about their effectiveness in other groups of children.

Furthermore, the overall effectiveness of implementation intentions may differ between children and adults due to developmental differences in self‐regulation abilities. On the one hand, effects have been demonstrated in patients with frontal lobe lesions (e.g. Lengfelder & Gollwitzer, 2001) and children with ADHD (e.g. Gawrilow & Gollwitzer, 2008), suggesting that implementation intentions may be effective even in populations with suboptimal self‐regulation abilities. On the other hand, findings in older adults suggest that there may be cognitive prerequisites, particularly in executive functions, for implementation intentions to be effective (Burkard et al., 2014; Hall et al., 2014). Especially younger children may lack these prerequisites as well.

Consequently, studies investigating the effects of implementation intentions in children often yielded inconsistent results. Gawrilow and Gollwitzer (2008) explored the effectiveness of implementation intentions in a Go/No‐Go task, testing the impact of furnishing a goal intention (‘I will refrain from pressing a key for pictures accompanied by sound!’) with a prespecified implementation intention (‘If I hear a tone, I will not press any key!’). Only children with ADHD benefited from the intervention, while those without ADHD did not. Other studies investigated effects of implementation intentions on health‐related behaviours like physical activity (Armitage & Sprigg, 2010) or the prevention of smoking (Higgins & Conner, 2003). In these instances, children are typically either prompted to internalize a given implementation intention (Armitage & Sprigg, 2010) or to choose one from a range of options or create their own (Higgins & Conner, 2003). The studies by Armitage and Sprigg (2010) and Higgins and Conner (2003) differed in the duration between the intervention's delivery and the outcome measurement (1 and 2 months, respectively). Armitage and Sprigg (2010) observed an effect of implementation intentions, whereas Higgins and Conner (2003) did not find a primary effect, but only effects involving moderators based on the Theory of Planned Behavior. Collectively, these findings suggest that there may be large heterogeneity in the effectiveness of implementation intentions in children that could be explained by factors such as the population under study, the specific details of the intervention and differences in methodology between studies. Identifying moderators that explain this heterogeneity is important for designing more effective interventions and uncovering the boundary conditions of implementation intentions in children.

### The present review and meta‐analysis

Our first main objective was to investigate the overall effectiveness of implementation intentions on goal attainment in children (age 12 or younger). Therefore, we integrated effect sizes of experimental studies that manipulated children's formulation of implementation intentions and tested the effect of this manipulation on children's goal attainment. We expected the evidence to support a positive effect of implementation intentions.

Second, we expected considerable heterogeneity in effect sizes due to differences between studies that are likely to moderate the effectiveness of the intervention. Specifically, studies may differ in: (a) the population (e.g. clinical vs. non‐clinical, child's age), (b) the intervention protocol (e.g. the level of assistance in formulating plans) or (c) the methodology (e.g. field study vs. laboratory setting, the time between providing the intervention and assessing the outcome). Although we took an exploratory approach to moderator analysis overall, we assumed directional effects for individual moderators. For example, we expected the intervention effect to be stronger when outcome measures are directly related to the then‐component of the plan rather than to a more global goal. An overview of all 18 examined moderators is displayed in Table S8 on the OSF project page.

## METHODS

The coding sheets, detailed results of the literature search, the R code and other supplementary materials have been shared on the Open Science Framework (https://doi.org/10.17605/OSF.IO/YD623).

### A priori power analysis

Previous meta‐analyses on the effects of implementation intentions provide little guidance for estimating the expected power. Only one previous meta‐analysis included a meaningful number of studies with children (*k* = 10), but they did not report separate statistics for these studies (Toli et al., 2016). The average sample size per study was 34 children. However, as the meta‐analysis only included clinical samples, we expected to include twice as many studies (*k* = 20) with larger sample sizes per study (average *N* = 60). We expected the effect size to be below the average effect size found in adult populations (*d* = .54), but close to the lower bound (i.e. *d* = .24; see Keller et al., 2020) and the heterogeneity to be moderate (*I* ^2^ = .50; see Bélanger‐Gravel et al., 2013; Silva et al., 2018; Malaguti et al., 2020).

We conducted an a priori power calculation with the metapower package, version 0.2.2 (Griffin, 2021). Based on *k* = 20 studies, on average *N* = 60 participants per study, an effect size of *d* = .24 and moderate heterogeneity of *I* ^2^ = .50, we estimated the power of the meta‐analysis to be .83. Figure 1a shows that 19 studies would be sufficient to achieve a power of .80. Power approaches 1 with 45 studies.

**FIGURE 1:**
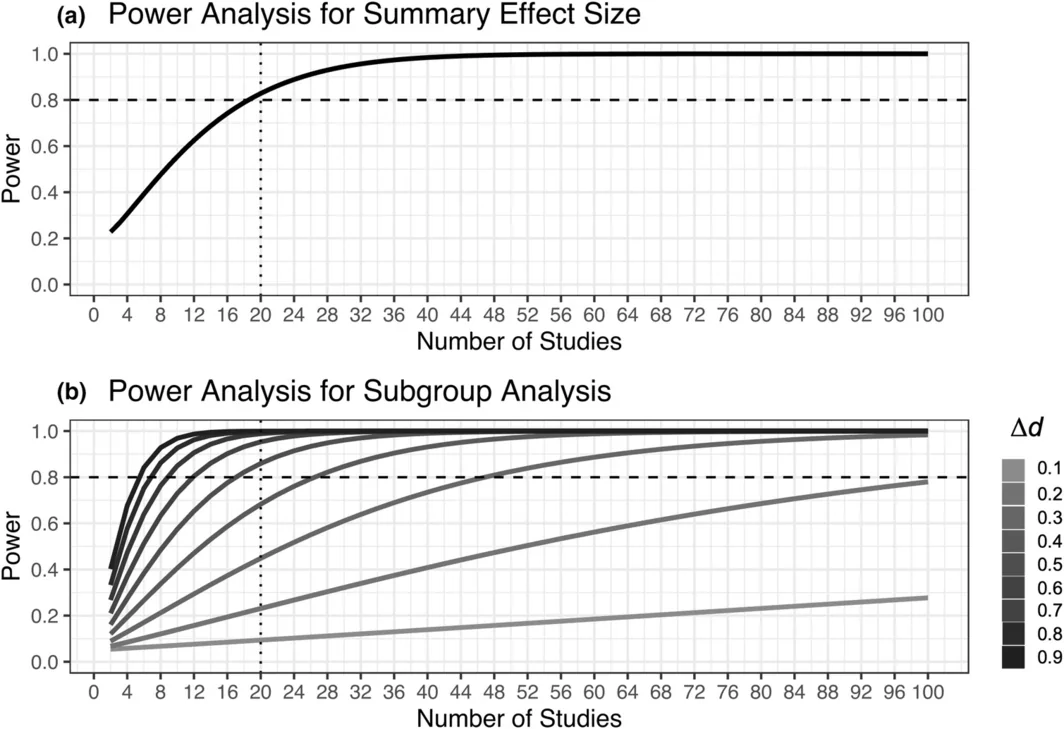
A priori power estimation for the summary effect size (a) and for the subgroup analysis (b). This figure was created with the ‘ggplot2’ package, version 3.5.2 (Wickham, 2016).

We also estimated power for the moderator analyses, based on the same parameters. We tried different effect sizes Δ*d* (i.e. the difference in effect size between subgroups), ranging from Δ*d* = .10 to Δ*d* = .90. As can be seen in Figure 1b, with 20 studies, a power of .80 can only be achieved if Δ*d* is at least .5. Therefore, we expected the statistical power to detect moderator effects to be rather low. Moderator analyses are thus considered exploratory.

### Eligibility criteria

A study was eligible for inclusion in the meta‐analysis if it fulfilled all of the following criteria: (1) The study included at least one group (between‐subject design) or condition (within‐subject design) *with* implementation intention and at least one group or condition *without* implementation intention, (2) the mean age of children at the time of the intervention was <13 years, (3) the report was written in English or German.

### Information sources

A systematic literature search was conducted in the following bibliographic databases in February 2024: PsycInfo, PSYNDEX (database of psychological publications from the German‐speaking countries) and PubMed. We chose these databases because they contain a thesaurus of subject terms that allow a standardized and reproducible search and cover both English and German literature as well as psychological and medical journals. Medical journals were included due to the many applications of implementation intentions in health contexts. To further identify unpublished doctoral dissertations, an additional search was conducted in ProQuest Dissertations & Theses Global. We sent a call to potential authors of relevant studies via the email distribution lists of the German Psychological Society, the Society of Personality and Social Psychology and the European Association of Social Psychology. Finally, the reference lists of all identified records were checked for additional studies, and authors of identified records were contacted for possible unpublished studies (see S1 on the OSF project page).

### Search strategy

To create the search string, we first listed 16 articles known to us that tested implementation intention effects in children under the age of 13 (see S2 on the OSF project page). Since the intervention is not represented by a subject term in any thesaurus, librarians must find a combination of the standardized thesaurus subject terms that best describe the content of the article and thus the concept of the intervention. Therefore, we searched the 16 articles in each database to identify the combination of standardized thesaurus subject terms that were used to describe the intervention (e.g. ‘intention’ combined with ‘self‐regulation’). In addition, we identified unstandardized keywords that authors typically use to describe the intervention (e.g. ‘implementation intention’, ‘if‐then plan’). We then combined the standardized subject terms and unstandardized keywords with keywords describing the age range. In PsycInfo, publications are systematically labelled with standardized terms for age groups; in PubMed, publications are not systematically labelled with terms for age groups; in PSYNDEX, there are no standardized terms for age groups. Therefore, we relied on standardized terms for age groups only in PsycInfo, whereas in PubMed and PSYNDEX, we described the age range using unstandardized keywords.

Search strings for each database were discussed with academic librarians. To validate the search strategy, we verified that all 16 articles were found. Minus syntactical differences between the different databases, the final search string was^1^:

(((SU “Intention” AND (SU (“Behavior Change” OR “Goals” OR “Self‐Regulation” OR “Planned Behavior” OR “Goal Orientation”))) OR (SU (“Goals” AND SU (“Self‐Control” OR “self regulation”))) OR SU “Action planning”) OR (“implementation intention*” OR “if then plan*” OR “planning intervention*” OR “action plan*” OR “coping plan*” OR “self‐reg* intervention*” OR “self‐control intervention*”)) AND (AG (Childhood OR Preschool Age OR School Age)).

The PRISMA flow diagram (Page et al., 2021) in Figure 2 displays the number of hits and the study selection process.

**FIGURE 2:**
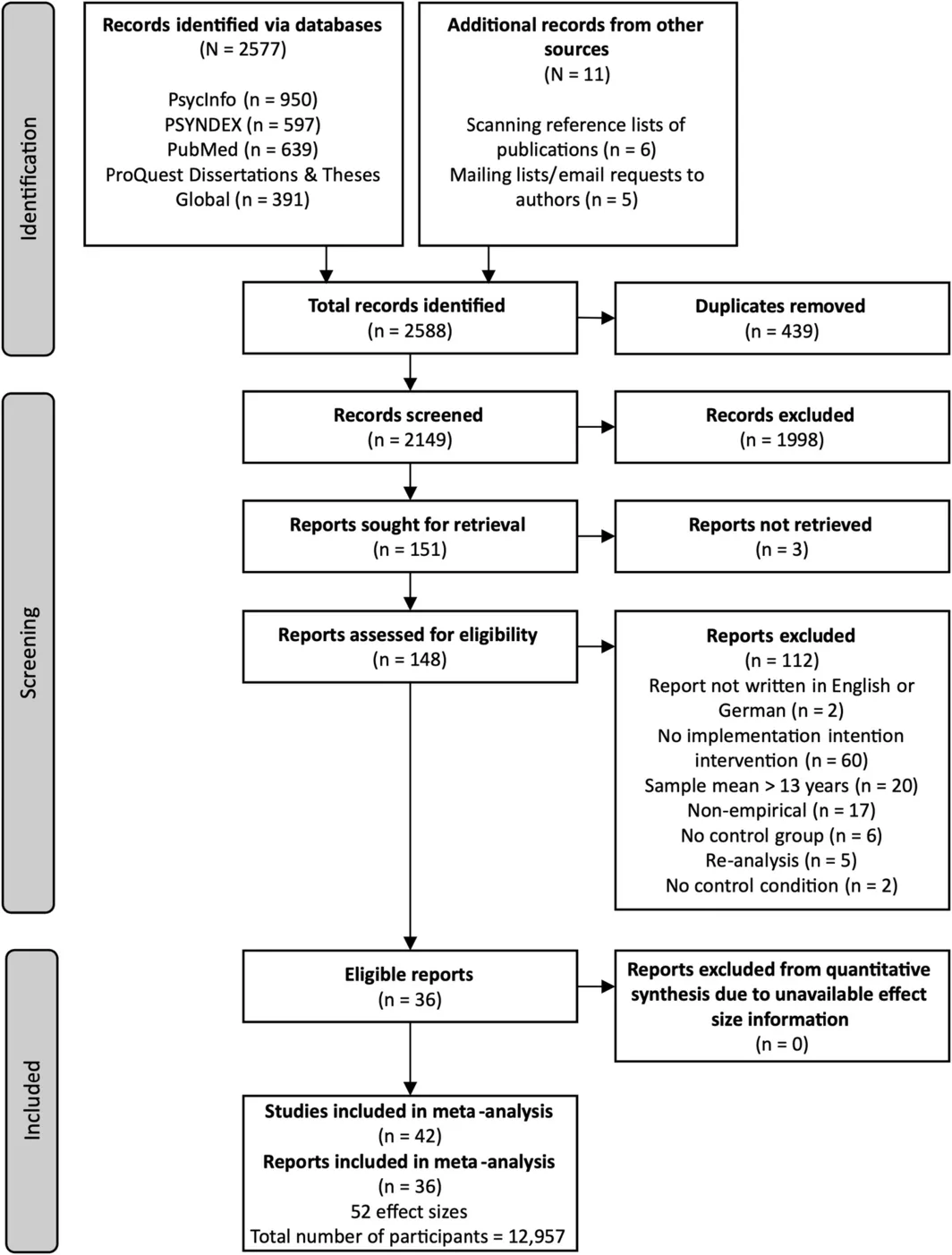
PRISMA flow diagram. The terms used in this figure follow Page et al. (2021). Report: document supplying information about a study. Record: title or abstract of a report indexed in a database. Study: investigation with a defined group of participants, interventions and outcomes. The number of studies exceeds the number of reports because some reports contained information on more than one study.

### Data management and selection process

The results of each database search were combined and duplicates were removed. Both authors independently screened the titles and abstracts of identified study records for eligibility. Subsequently, both authors independently screened the full texts of the remaining studies for the final inclusion; disagreements were resolved through discussion. Reasons for exclusion were documented (e.g. wrong age range; see S4 on the OSF project page). If a study did not offer all relevant information to determine inclusion or exclusion, we contacted the authors of the study.

#### Data collection process

For the final data extraction from the eligible studies, each of the two raters independently entered the descriptive information about the studies and the information needed to calculate effect sizes in the coding sheets (see S5 and S6 on the OSF project page). We resolved disagreements by discussion. The coding sheets were piloted and optimized by coding a total of 10 studies on implementation intention effects in adults (Achtziger et al., 2008; Adriaanse et al., 2009; de Nooijer et al., 2006; de Vet et al., 2009; Latimer et al., 2006; Luszczynska, 2006; Prestwich et al., 2009; Prestwich et al., 2010; Rodrigues et al., 2013; Smith et al., 2022).

We contacted authors of studies that lacked necessary information on study characteristics or study results. The authors of one study provided the data set together with the analysis file, so that we could calculate the effect sizes from the data (Duckworth et al., 2013). We documented whether effect sizes were calculated or obtained from the published record. The filled‐out coding sheets agreed upon by both authors have been made available on the OSF project page.

### Data items

If a study reported effects for more than one dependent variable, we only coded the dependent variable that most closely reflected the achievement of the action specified in the implementation intention. As a hypothetical example, a study might ask children to internalize the implementation intention: ‘If someone offers me cake, then I will say ‘no’ and eat an apple instead’. The study might then report effects on different dependent variables, such as a score indicating how many apples children ate in the following 30 days, children's BMI after 30 days and a score indicating to what extent children view themselves as healthy eaters. In this case, we would have included only the effect on the first score (i.e. the number of apples eaten) in the meta‐analysis because it best reflects the implementation of the behaviour specified in the plan. If a study reported effects on scores from different scales that all measured the same construct (e.g. self‐control), we used the score with the best psychometric properties. We expected that, for most studies, one dependent variable can be identified that best reflects the implementation of the plan, because implementation intentions usually specify only one specific action. For this variable, we coded the information needed to calculate the effect size (e.g. *N*, *M* and *SD* for each experimental and control group).

Regarding study characteristics, we coded global study information (e.g. language of publication), correspondence with study authors (if applicable), sample information (e.g. mean age), design information (e.g. between‐ or within‐subject design), information on the outcome variables (e.g. description of the primary outcome) and information on each intervention and control group (e.g. description of the instruction).

### Synthesis methods

The meta‐analysis integrated both continuous dependent variables (e.g. scores on a self‐regulation scale) and categorical dependent variables (e.g. goal achievement). We expected that the vast majority of studies use the standard between‐subjects design, comparing independent groups of participants with and without implementation intention (Gollwitzer, 1999). We also found two eligible studies with three independent effect sizes that used a within‐subject design (Paul et al., 2007; Schaaf et al., 2025). Leaving these effect sizes out of the analysis would only have led to a very minor change in the combined effect size. We therefore integrated results from between‐ and within‐subject designs to achieve higher power.

We used R, version 4.4.2 (R Core Team, 2024), together with RStudio, version 2025.9.2.418 (Posit team, 2025) and the ‘meta’ package, version 8.2‐0 (Balduzzi et al., 2019) to perform the analyses. We used a random‐effects model because we expected substantial heterogeneity between studies.

We report Hedges' *g* as the measure of effect size, using the approximated bias correction factor (Hedges & Olkin, 1985). We re‐coded the means of the dependent variable where necessary so that higher values indicated better performance. The effect size estimates are based on the descriptive statistics reported in the manuscript (*M* and SD for continuous outcome variables; percentages or counts for categorical outcome variables). If descriptive statistics were not reported in the manuscript, we used test statistics (e.g. *t* and *df*) to calculate effect sizes (see S7 on the OSF project page for a detailed account of effect size calculation). All values for the meta‐analysis are documented in the study results coding sheet (see S6 on the OSF project page).

If a study included multiple experimental groups with implementation intention or multiple control groups, we calculated the combined mean and the combined standard deviation across experimental groups and control groups, respectively, to create a single comparison per study (see Higgins et al., 2022, Chapter 6.5.2.10).

We used several methods to assess the extent to which the results of the meta‐analysis may have been influenced by individual studies. We defined outliers as effect sizes whose 95% confidence interval did not overlap with the 95% interval of the pooled effect (Viechtbauer & Cheung, 2010). For the influential case analysis, we used the leave‐one‐out method to detect to what extent each study influenced the overall result of the meta‐analysis (Viechtbauer & Cheung, 2010). We present a Baujat plot (Baujat et al., 2002) showing the influence of each study on the Cochrane *Q*‐test for heterogeneity and on the pooled effect size. Influence diagnostics for each study *k* can be found in Section 6 of the analysis script on the OSF project page. These diagnostics include (a) the externally standardized residual *t*_*k*_ (i.e. the deviation from the pooled effect size), (b) the DFFITS_k_ value (Viechtbauer & Cheung, 2010), which indicates by how many SD the pooled effect changes if study *k* is removed, (c) Cook's distance *D*_*k*_, (d) the covariance ratio CovRatio_*k*_ (i.e. the variance of the effect without study *k* divided by the variance of the pooled effect), (e) the leave‐one‐out *Q*_*k*_ statistic, (f) the hat_k_ value and the study weight_*k*_. A study was described as an influential case if it fulfilled at least one of three criteria: (1) DFFITS_*k*_ >3√1/(*k* − 1), (2) *D*_*k*_ >0.45, (3) hat_*k*_ >3(1/*k*).

### Risk of bias assessment

The authors independently assessed the risk of bias in the included studies using the Cochrane Risk of Bias Tools, which were suitable for each study (RoB 2.0, RoB 2 crossover, ROBINS‐I; Eldridge et al., 2016; Sterne et al., 2016, 2019). The outcome of interest (selected as described in the Data Items section) was assessed for risk of bias arising from the randomization process, deviations from the intended interventions, missing outcome data, measurement of the outcome and selection of the reported result. Bias in the domain ‘selection of the reported result’ was assessed in light of the fact that the outcome selected for this meta‐analysis may not be the same as the primary outcome selected by the study authors. Judgements of low risk, some concerns or high risk were made using the signalling questions within each domain. Disagreements between reviewers were resolved by discussion and consensus.

### Reporting bias assessment

To assess the risk of reporting bias in the meta‐analytic result, the following analyses have been performed. First, we present a funnel plot together with tests of its asymmetry to detect small‐study effects. We compared different estimation methods and report if they lead to different conclusions (Begg & Mazumdar, 1994; Egger et al., 1997; Pustejovsky & Rodgers, 2019; Thompson & Sharp, 1999). We calculated a bias‐corrected pooled effect using the PET‐PEESE method (Stanley, 2007; Stanley & Doucouliagos, 2014). In addition, we used the three‐parameter selection model (3PSM) to estimate the average true underlying effect, the heterogeneity of the random effect sizes and the probability that a non‐significant effect enters the literature (Carter et al., 2019). Finally, we used methods to detect publication bias based on *p*‐values: *p*‐curve (Simonsohn et al., 2014a, 2014b, 2015), *p*‐uniform* (van Aert & van Assen, 2021) and *z*‐curve analyses (Bartoš & Schimmack, 2022).

### Moderator analyses

We performed an exploratory moderator analysis to explain between‐study heterogeneity, focusing on 18 potential moderators. Examples include the mean age of the sample, the fit of the plan with the primary outcome and the duration between creating the implementation intention and the measurement of the outcome. Table S8 (on the OSF project page) displays the full list of considered moderators, the rationale to include these moderators and the expected direction of effect.

We used MetaForest (van Lissa, 2020) to address the problem of limited studies and lack of statistical power for moderator analyses. MetaForest is an exploratory, data‐driven technique that adapts the random forest approach to meta‐analysis. MetaForest has the added advantage of capturing nonlinear relationships between moderators and effect sizes as well as higher level interactions. We conducted the analysis in accordance with the recommendations of van Lissa (2020), including fine‐tuning of the model. MetaForest cannot determine which moderators are significant, but it does allow for the ranking of moderators by their relative importance. This exploratory moderator analysis was followed by meta‐regression, focusing only on the relevant moderators.

### Sensitivity analyses

We conducted several sensitivity analyses to test the robustness of the meta‐analytic results. Specifically, we tested five changes that might affect the results of the meta‐analysis. First, we tested how results change if outliers and influential cases are removed. Second, we repeated the analysis excluding studies with a high‐risk judgement. Third, we tested whether results change when different but comparable outcomes were included (e.g. response times instead of error rates in a cognitive task where the implementation intention emphasized both speed and accuracy). We had planned to re‐run the meta‐analysis with all possible combinations of effect sizes. However, since the number of possible combinations was very large (2^18^), we instead drew a random sample of 10% from all possible combinations and re‐ran the meta‐analysis with those. Fourth, if the study originally reported analyses that controlled for variables (e.g. demographic variables), we coded this information and repeated the meta‐analysis using these controlled effect sizes instead of the effect sizes calculated from the descriptive data. Fifth, we checked for effects of selecting different time intervals between the intervention and the outcome (i.e. using the last outcome assessment instead of the first outcome assessment).

## RESULTS

### Summary of studies

We included 52 effect sizes from 42 studies (see Appendix A for an overview of included studies). In eight of these studies, both clinical (children with ADHD) and non‐clinical samples were examined, each providing an experimental and a control group (Boscher, 2006; Gawrilow & Gollwitzer, 2008, Study 1; Gawrilow et al., 2011a, Study 1 and Study 2; Gawrilow et al., 2011b, Study 2; Paul et al., 2007; Schwantje, 2005; Stumpf, 2004). Because outcomes were reported separately for each group and sample, we coded the effect sizes separately, resulting in 32 effect sizes. Taken together, there were 12 effect sizes from clinical samples (11 ADHD, 1 repaired cleft lip and/or palate) and 40 from non‐clinical samples. In two further studies, separate effect sizes were computed for younger and older children (Kretschmer‐Trendowicz et al., 2021, Study 1 and Study 2).

Sample sizes across studies ranged from *n* = 13 to *n* = 5974 (Median = 47.5, *M* = 295.83, *SD* = 838.29). The total number of participants included in the present meta‐analysis is 12,957. Participants were children, with a mean age of 10.67 years (range: 4.5–12.99 years). On average, 47.70% of participants were female (range = 0 % to 100 %).

The large majority of effect sizes were based on samples from Germany (*n* = 31), followed by the United States (*n* = 7) and England (*n* = 5). The studies were published between 1975 and 2025 (median publication year = 2011). Of the 52 effect sizes, 44 were drawn from peer‐reviewed journal articles, seven from Diploma theses and one from a PhD thesis.^2^

Thirty‐two effect sizes were from studies investigating the effects of implementation intentions on task performance in laboratory settings, typically measuring delay of gratification, executive functions or prospective memory. Of the 20 field studies, 10 effect sizes came from studies targeting physical health behaviours, including oral health (*n* = 3), smoking (*n* = 3), physical activity (*n* = 2) and healthy eating (*n* = 2). Seven studies focused on academic goals, two on interpersonal goals and one on coping with advertising. Four studies combined implementation intentions with mental contrasting. In four studies, implementation intentions were part of a larger intervention (e.g. including other information, behaviour training or behaviour change techniques).

### Overall effectiveness of implementation intentions

The synthesis of all 52 effect sizes confirmed a significant small to medium effect of implementation intentions in children (*g* = 0.31, 95% CI [0.21; 0.41], *p* < .001, *k* = 52; see Figure 3). Only three of the 52 effect sizes stem from within‐person comparisons; thus, no meaningful quantitative comparison was possible. The effects in the within‐person studies were rather small, namely, 0.08, 0.11 and 0.28 (overall *g* = 0.12, 95% CI [0.02; 0.21], *p* = .020, *k* = 3). Removing these three studies from the meta‐analysis would thus result in a slightly higher overall effect estimate (*g* = 0.33, 95% CI [0.22; 0.44], *p* < .001, *k* = 49).

**FIGURE 3:**
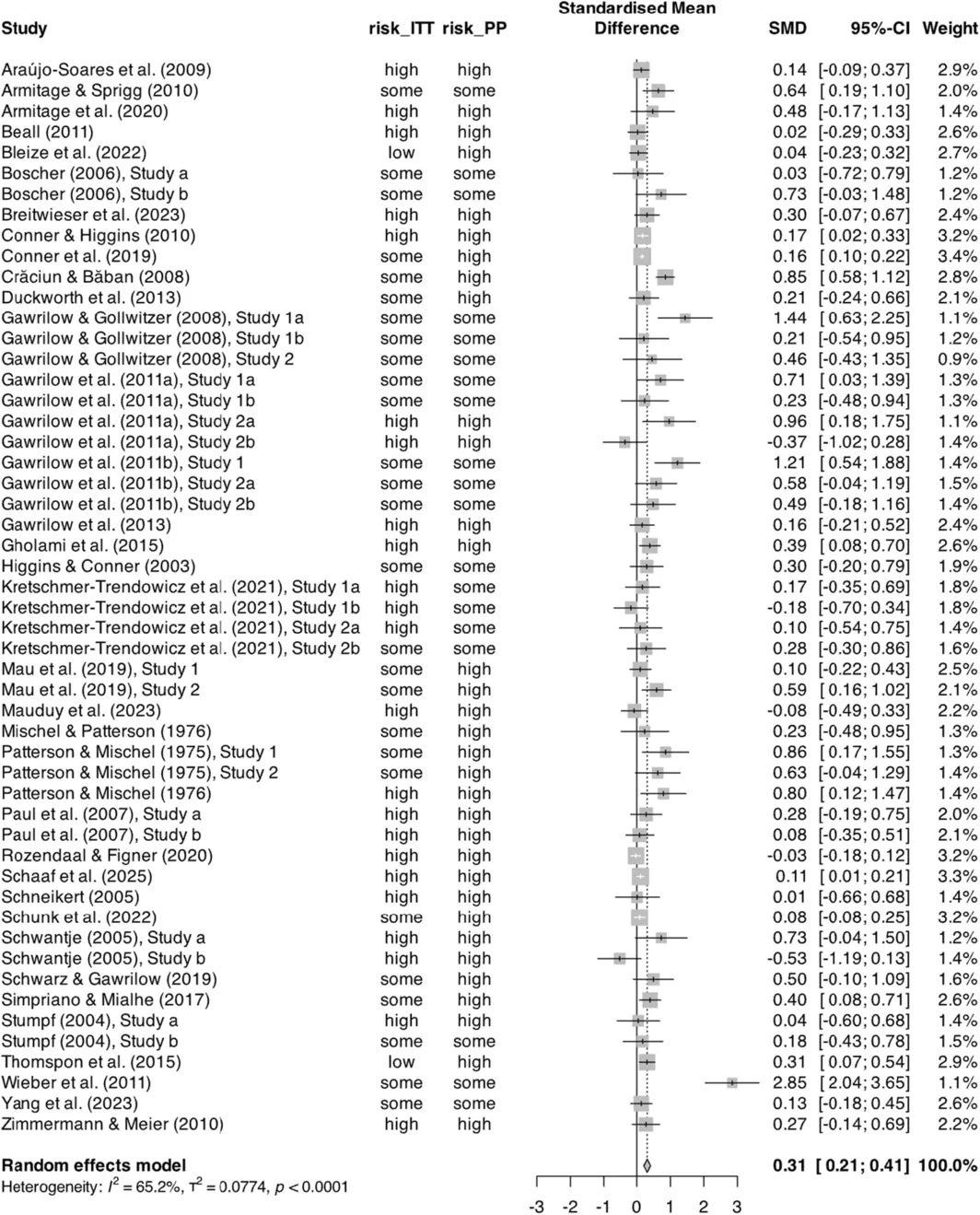
Forest plot. risk_ITT = risk assessment for an intention‐to‐treat analysis; risk_PP = risk assessment for a per‐protocol analysis. This figure was created using the forest function from the ‘meta’ package, version 8.2‐0 (Balduzzi et al., 2019).

There was substantial heterogeneity in the size of the effects across the 52 effect sizes (*Q*(*df* = 51) = 146.47, *p* < .001; *I* ^2^ = 65.2% [53.3%; 74.0%]). The Baujat plot (Figure 4) shows that some studies had a very high influence on the pooled results. Six studies (Crăciun & Băban, 2008; Gawrilow & Gollwitzer, 2008, Study 1a; Gawrilow et al., 2011b, Study 1; Rozendaal & Figner, 2020; Schwantje, 2005, Study b; Wieber et al., 2011) were identified as outliers since the 95% CI of their effect size lay outside the 95% CI of the pooled effect. Based on the influence diagnostics, Crăciun and Băban (2008) and Wieber et al. (2011) were identified as influential cases due to their strong contribution to the heterogeneity of the effect sizes. At 2.85, the study by Wieber et al. (2011) had the largest effect size. Although not the second largest, the effect size of 0.85 in the study by Crăciun and Băban (2008) was also considerably larger than the pooled effect. This study had a comparatively large sample size and thus influence on the overall result.

**FIGURE 4:**
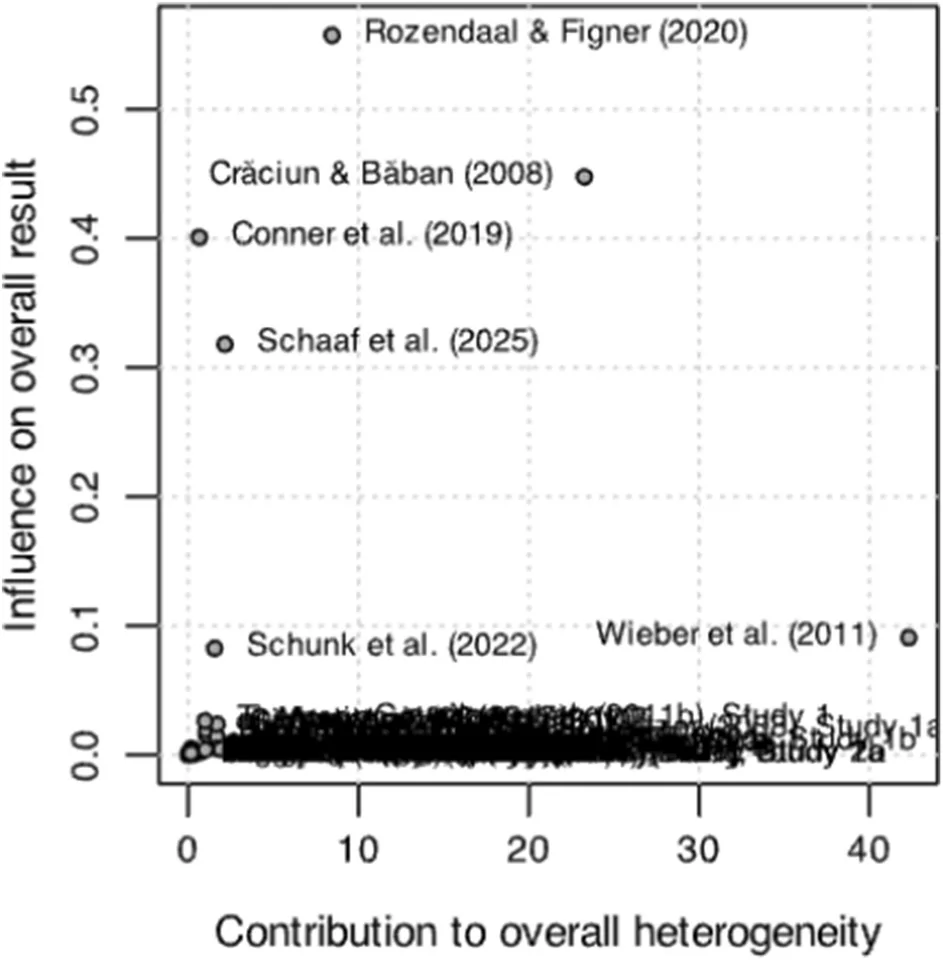
Baujat plot. This figure was created using the baujat function from the ‘meta’ package, version 8.2‐0 (Balduzzi et al., 2019).

### Risk of bias

The included studies differed in terms of the implementation intention effects they examined. Some studies (e.g. Armitage & Sprigg, 2010; Conner & Higgins, 2010) explicitly stated that they were interested in the intention‐to‐treat effect, that is, the effect of assigning children to the implementation intention intervention regardless of their adherence to the intervention. Other studies excluded children after randomization, for example, because they failed a manipulation check (e.g. Kretschmer‐Trendowicz et al., 2021, Studies 1a, 1b, and 2a). These studies thus focused on the per‐protocol effect, that is, the effect of implementation intentions among children who sufficiently understood the intervention. However, most studies did not explicitly indicate whether they followed an intention‐to‐treat or a per‐protocol approach. Therefore, we decided to assess both the risk of bias for an intention‐to treat analysis and for a per‐protocol analysis.

Regarding the intention‐to‐treat analysis, two of the 52 effect sizes were rated as low risk of bias, 27 had some concerns and 23 were judged as high risk of bias. Ratings of some concerns were mainly due to missing preregistration of outcomes and analyses plans, while ratings of high risk of bias were often based on baseline differences between groups, potentially selective dropout rates, or the exclusion of participants after randomization. With regard to the per‐protocol analysis, risk of bias was generally higher: No effect size was rated as low risk of bias, 19 had some concerns and 33 were considered of high concern, primarily due to insufficient reporting of whether children created implementation intentions of sufficient quality, or due to reported problems with the quality of child‐created plans (e.g. Beall, 2011; Schaaf et al., 2025). Overall ratings of risk of bias for each study are displayed as part of Figure 3. Detailed information on single risk of bias criteria are presented in Supplement S9 on the OSF project page.

### Reporting bias

We assessed small study effects using a funnel plot (Figure 5). The plot revealed asymmetry, with three effect sizes showing high standard errors and strong statistical significance of positive effects. In contrast, more precise estimates tended to cluster on the left side of the pooled effect. The asymmetrical pattern was confirmed by the significant Egger's regression test (*t*(50) = 3.15, *p* = .003). Additional methods for detecting funnel plot asymmetry yielded similar results (all *p* < .05; see section 6.2 of the meta‐analysis.html output on the OSF project page). These results indicate that there is a possibility of small study effects.

**FIGURE 5:**
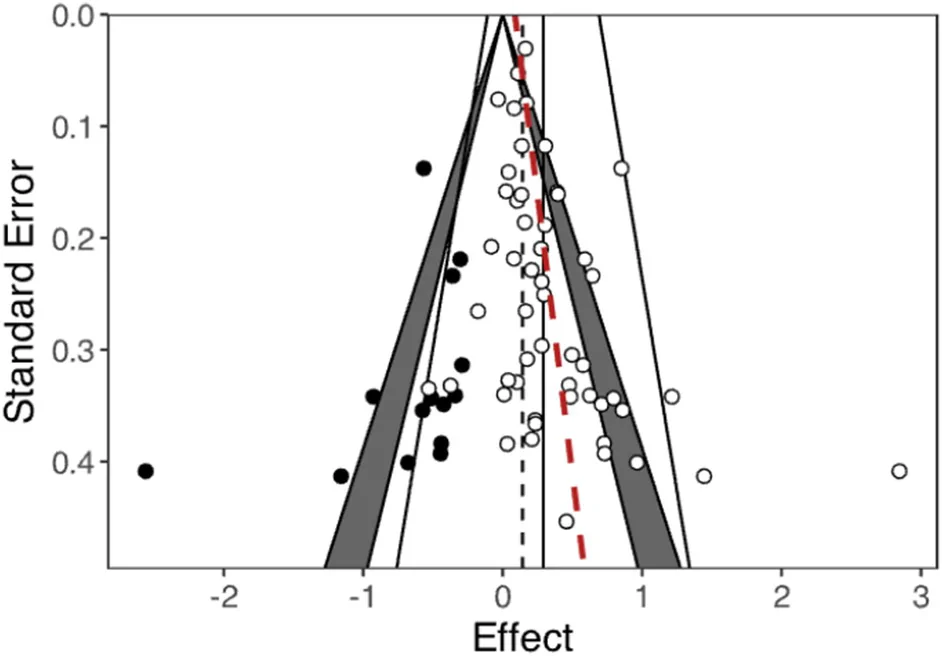
Funnel plot. The contour‐enhanced funnel plot includes confidence contours at the .05 > *p* > .01 level (grey area). The plot also shows Egger's regression line (red dashed line) and 14 studies imputed by the trim and fill method (black filled dots). This figure was created using the vizfunnel function from the ‘metaviz’ package, version 0.3.1 (Kossmeier et al., 2020).

We used the PET‐PEESE method to estimate a bias‐corrected version of the pooled effect. The PET model had a significant positive intercept (*b* = 0.10, *p* = 0.026). Therefore, we used the intercept of the PEESE model as the true effect size estimate (*b* = 0.15, *p* < .001). This result differed from the estimated true average effect size using the three‐parameter selection model. The estimated effect size was 0.38 (*p* < .001), with substantial heterogeneity among the studies (LRT(1) = 20.95, *p* < .001). The test for selection bias indicated no significant evidence of selective reporting (LRT(1) = 0.96, *p* = .327). Specifically, the estimated probability of inclusion for studies with non‐significant results (.025 < *p* ≤ 1) did not differ significantly from that for significant results (estimate = 1.71, 95% CI [0.00, 3.53], *p* = .446). Thirty‐six of the effect sizes were not significant compared to 16 that were significant. The *p*‐curve, *p*‐uniform* and *z*‐curve analyses arrived at similar adjusted effect estimates, comparable to those obtained from the three‐parameter selection model (*p*‐curve: *d* = 0.35; *p*‐uniform*: *d* = 0.39, *z*‐curve: *d* = 0.35). These estimates reflect the effect magnitude implied by the observed distribution of statistically significant results under selection. Note, however, *p*‐curve and *p*‐uniform* are generally not considered robust when the between‐study heterogeneity is higher than *I* ^2^ = 50% (van Aert & van Assen, 2021). The observed discovery rate (ODR = 0.35, 95% CI [0.22, 0.49]) exceeded the expected discovery rate (EDR = 0.23, 95% CI [0.05, 0.68]), indicating that statistically significant findings are likely overrepresented relative to what would be expected based on the estimated effect‐size distribution and statistical power (see *z*‐curve figure in section 6.8 of the meta‐analysis.html output on the OSF project page).

### Moderator effects

Most of the variables we considered as moderators were heavily skewed, indicating that there are typical methods of delivering implementation intentions to children and common study designs. For example, the large majority of studies used if and then components that described external (rather than internal) cues and behaviours. Thirty‐three studies used implementation intentions that were predetermined by the experimenter; only 14 studies asked children to formulate their own plans, and five studies let children create plans by choosing from provided examples. Furthermore, 40 of the 52 effect sizes are based on non‐clinical samples. A full overview of all candidate moderators is provided in Appendix B.

To identify relevant moderators, we first performed the random‐effects weighted MetaForest analysis. As an initial step, we conducted variable pre‐selection with 20,000 iterations and replicated 100 times with a recursive algorithm. We retained only the three moderators that had positive variable importance in more than 50% of replications: mean age, type of sample (clinical vs. non‐clinical) and number of plans (one vs. more than one). These moderators were kept and carried forward for model optimization. The cross‐validated explained variance was *R* ^2^_cv_ = 0.40; the out‐of‐bag explained variance was *R* ^2^_oob_ = 0.01. The relative importance of included moderators in the final model is displayed in Figure 6.

**FIGURE 6:**
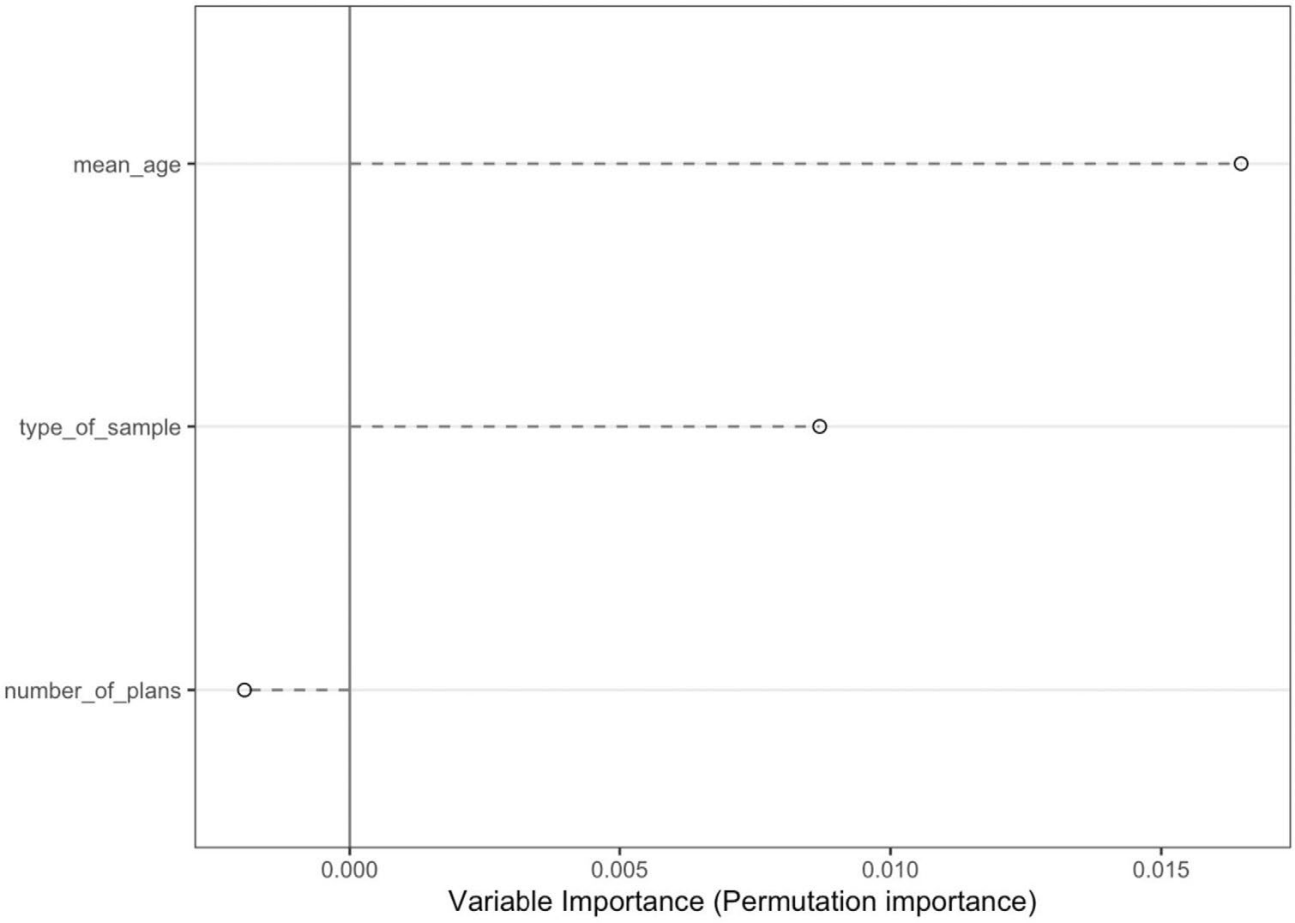
Variable importance plot. This figure was created using the VarImpPlot function from the ‘MetaForest’ package, version 0.1.5 (van Lissa, 2020).

Finally, we included the three most important moderators into a linear meta‐regression (see Table 1). Mean age emerged as a significant predictor of effect size, indicating that intervention effects decreased as the average age of participants increased. All else being equal, each additional year in mean age was associated with a reduction in effect size of approximately 0.05 Hedge's *g* units. In other words, implementation intention interventions were more effective in studies involving younger children. Type of sample was not significant in a two‐sided test (*p* = .075). The trend was in the direction of effect sizes being higher in studies with clinical samples compared to non‐clinical samples.

**TABLE 1:** Results of the meta‐regression.

Variable	Estimate	SE	95% confidence interval	*p*‐value
Intercept	1.13	0.27	[0.59, 1.66]	<.001
Mean age	−0.05	0.02	[−0.10, −0.01]	.022
Type of sample (non‐clinical)	−0.25	0.14	[−0.52, 0.02]	.075
Number of plans (more than one)	−0.14	0.10	[−0.35, 0.06]	.170

*Note*: Due to a missing value on mean age, one study (Beall, 2011) was not part of the meta‐regression. For the variable fit of outcome, the reference category was the category ‘low’. For the variable plan creation, the reference category was ‘self‐created’.

Our pre‐registration stated that we would omit the variables *action* versus *coping planning*, *type of if‐component* and *type of then‐component* from the MetaForest analysis because we anticipated substantial missing data. In practice, missingness was modest (4, 6 and 10 cases, respectively). Adding these moderators left the pattern of results unchanged.

We also repeated the moderator analyses by removing the two studies that were identified as influential cases (Crăciun & Băban, 2008; Wieber et al., 2011). Here, *type of sample* was a significant predictor (*b* = −0.38; *p* = .002). *Mean age* remained a significant negative predictor (*b* = −0.04, *p* = 0.05). In addition, the predictor *plan format* reached significance. Interventions that guided children with pre‐structured sheets to form implementation intentions outperformed those using the standard if–then sentence structure (*b* = 0.32, *p* = .014). Note, however, that only four studies used pre‐structured sheets. The moderator *plan creation* was not significant in a two‐sided test; the tendency was for experimenter‐provided plans to be more effective than self‐created plans (*b* = 0.44, *p* = 0.98). The predictors *fit of the plan to the outcome*, *number of plans*, *setting* (laboratory vs. field) and *time between plan creation and first outcome assessment* also had possible variable importance in this analysis but did not reach significance in the meta‐regression.

Because the variable *experimenter assistance* (i.e. whether or not the experimenter helped children during planning) is only relevant when children generate their own plans, we analysed this moderator within the 14 studies with self‐created plans. Due to the low number of studies, we could not conduct the full MetaForest analysis. However, experimenter assistance was not significantly associated with effect size in these 14 studies (*b* = 0.11, *p* = .703).

In the 38 studies where plans were provided by the experimenter, we repeated the full moderator analysis by including two additional variables that indicated whether the if‐ or then‐component of the provided plans had been derived from pilot data. *Type of sample* remained the only significant predictor in the final meta‐regression (*b* = −0.57, *p* = .035).

Taken together, *mean age* emerged as a robust, significant moderator. When influential cases were removed, *type of sample* and *number of plans* also showed moderating effects. Furthermore, based on the results, two moderators warrant further investigation: *plan format* (prestructured sheet vs. if–then sentence) and the *method of plan creation* (self‐created, based on provided examples, or fully provided by the experimenter).

### Sensitivity analyses

First, we tested how the results of the meta‐analysis change when excluding outliers and influential cases. Excluding the seven studies identified as outliers from the meta‐analysis resulted in a considerably lower effect estimate with a pooled effect size of *g* = 0.18 (95% CI [0.14; 0.22], *p* < .001, *k* = 46). Excluding the outliers also lowered the between‐study heterogeneity considerably (*Q*(45) = 49.43 with *p* = .301; *I* ^2^ = 9.0%). When only excluding the two studies identified as influential cases, the pooled effect size was *g* = 0.22 (95% CI [0.15; 0.28], *p* < .001, *k* = 50, *Q*(49) = 79.97 with *p* = .003; *I* ^2^ = 38.7%).

Second, we tested how the results would change when only including effect sizes with a low risk of bias judgement. We coded risk of bias both for the effect of assignment to the intervention (i.e. intention‐to‐treat) and for the effect of adhering to the intervention. Because there were only two effect sizes with a low risk of bias judgement, we also included effect sizes with some concerns. For the effect of assignment to the intervention, there were 29 effect sizes with low or some concerns. The pooled effect size across these studies was *g* = 0.47 (95% CI [0.31; 0.64], *k* = 29; *Q*(28) = 107.32 with *p* < .001; *I* ^2^ = 73.9%). For the effect of adhering to the intervention, there were 19 effect sizes with low or some concerns. The pooled effect size across these studies was *g* = 0.52 (95% CI [0.24; 0.80], *k* = 19; *Q*(18) = 62.02 with *p* < .001; *I* ^2^ = 71.0%).

Third, we tested how the results would change if effect sizes were calculated based on alternative outcome variables. Importantly, we did not include all of the dependent variables reported in a study but only those that fit the target of the implementation intention equally well. Such alternative outcome variables were identified for 18 of the 40 effect sizes. We had planned to re‐run the meta‐analysis with all possible combinations of effect sizes. However, since the number of possible combinations is very large (2^18^ = 262,144), we instead drew a random sample of 10% from all possible combinations and re‐ran the meta‐analysis with those. The average pooled effect across all iterations was *g* = 0.30 (*SD* = 0.02, range: 0.24–0.36, average *I* ^2^ = 60.26%). As this is very close to the original meta‐analytic result, we see no sign of the effect estimate being biased by the choice of outcome variable.

Fourth, we tested how the results would change if the effect sizes were calculated not based on the descriptive statistics reported in the studies, but on the basis of statistical tests that controlled for other variables, such as baseline differences between groups or nested data structures. Such alternative effect sizes could be calculated for nine out of the 52 effect sizes. Across the 2^9^ = 512 iterations of the meta‐analysis, the average pooled effect was *g* = 0.32 (SD = 0.01, range: 0.31–0.33, average *I* ^2^ = 65.31%).

Fifth, we tested how the results would change if effect sizes were calculated based on the last assessment of the outcome variable rather than the first assessment after the intervention was delivered. The average time between intervention and the first outcome assessment was 67.04 days (SD = 283.11 days, range: 0–1461 days). Ten out of the 52 effect sizes could be re‐calculated based on later outcome assessment. When replacing all 10 of these effect sizes at once, the average time between intervention and assessment changed to 88.25 days (SD = 286.59 days, range: 0–1461 days). Across the 2^10^ = 1024 iterations of the meta‐analysis, the average pooled effect was *g* = 0.34 (SD = 0.01, range: 0.32–0.37, average *I* ^2^ = 71.34%).

In addition to the pre‐registered sensitivity analyses, we conducted an exploratory sensitivity analysis in which we excluded all effect sizes that were extracted from student theses (grey literature). Some authors have argued that student theses often report studies that are fraught with errors, underpowered and not conducted with the same level of competence as studies led by experienced researchers. According to this argument, including these effect sizes in a meta‐analysis could introduce a bias towards lower effect estimates (Cunningham & Baumeister, 2016). The present meta‐analysis includes seven effect sizes from student Diploma theses, written between 2004 and 2006. Removing these effect sizes resulted in a pooled effect of *g* = 0.33 (95% CI [0.22; 0.44], *k* = 45; *Q*(44) = 137.34 with *p* < .001; *I* ^2^ = 68.0%).

## DISCUSSION

This meta‐analysis provides robust evidence that implementation intentions can support children in achieving a range of desirable outcomes, including improvements in academic, health‐related and interpersonal behaviours as well as executive functioning. Across all studies, implementation intention interventions had a small to medium effect (*g* = 0.31, 95% CI [0.21; 0.41]). This effect should be interpreted in light of the substantial heterogeneity observed across studies, highlighting the need to identify the conditions under which implementation intentions are most effective and to examine how participant characteristics, intervention delivery and contextual factors moderate their impact.

Sensitivity analyses further revealed that the estimated effect size varied considerably depending on which studies were included. Excluding studies characterized by small sample sizes and unusually large effects that were flagged as outliers decreased the estimated effect size to *g* = 0.18. This suggests that small study effects may have inflated the overall estimate, a conclusion also supported by the asymmetry present in the funnel plot. Conversely, when studies with a high risk of bias were excluded, the effect size increased to *g* = 0.52, indicating that high‐quality studies achieved larger effect sizes.

### Comparisons with previous meta‐analyses

Our effect estimate is broadly consistent with those reported in previous meta‐analyses. At the time of registering this report, Toli et al.'s (2016) work was the only meta‐analysis that included any child samples. At first glance, our effect estimate appears considerably lower than the large effect (*d* = 0.99) reported by Toli et al. for implementation intentions in populations with mental health problems. However, a closer examination reveals greater alignment: when averaging only the eight^3^ effect sizes based on child samples from their study, the estimate is considerably lower (*d* = 0.57). This closely mirrors the effect size in our meta‐analysis when averaging across those for children with ADHD or at higher likelihood of having ADHD (*g* = 0.55).

Between the first and second stage of this registered report, a new meta‐analysis by Sheeran et al. (2024) was published. Their analysis included 642 effect sizes overall, 31 of which came from samples of children under the age of 12 that yielded an average effect size of *d* = 0.43 (95% CI [0.27, 0.59]). The present meta‐analysis extends this work by including 52 effect sizes from child samples. This increase reflects both our broader inclusion criteria and a more up‐to‐date search. Unlike Sheeran et al., we did not exclude studies in which implementation intentions were part of a multicomponent intervention. While such studies may confound attribution of effects to a single mechanism, they are practically relevant and may reflect real‐world applications of interventions in educational and clinical contexts. Furthermore, Sheeran et al.'s inclusion period spanned 1994–2019, while we included 14 effect sizes published after 2019 as well as four effect sizes from studies conducted in the 1970s by Walter Mischel and Charlotte Patterson. Although the concept of implementation intentions is typically attributed to Gollwitzer (first published in the 1990s), we found earlier studies that used comparable if‐then planning strategies with children and were cited in foundational implementation intention research (e.g. Wieber et al., 2011). These studies met our eligibility criteria and were therefore included. Finally, we also included grey literature (e.g. diploma theses). Together, these additions provide a comprehensive and up‐to‐date synthesis of implementation intention interventions for children.

Although our pooled effect size falls within the 95% confidence interval reported by Sheeran et al. for child samples, it represents a more conservative estimate. One contributing factor is the larger proportion of recent studies in our sample, which tend to report smaller effect sizes (see Appendix C). This trend may reflect changes in study design, sample characteristics or publication practices over time. For example, all included studies involving children with ADHD (a population for whom implementation intentions appear particularly effective) were conducted between 2004 and 2011. Furthermore, Sheeran et al. report strong evidence of publication bias in their meta‐analysis. When applying robust estimation methods, their overall effect size dropped from 0.36 to 0.15; however, they did not provide a separate corrected estimate for child samples. In our analysis, we found evidence of small study effects, and the overall effect size decreased when statistical outliers were excluded. While the presence of a substantial number of non‐significant effect sizes suggests that non‐significant findings are not entirely absent from the literature, our z‐curve results indicate that statistically significant findings may still be overrepresented relative to what would be expected based on the estimated distribution of true effects and statistical power. Taken together, these findings suggest that, while the effects of implementation intentions in children are robust, they could be somewhat smaller than previously assumed.

### Moderators of implementation intention effects in children

#### Implementation intentions are more effective in younger children and children with ADHD

A key contribution of this meta‐analysis is the focused examination of moderators in studies with child samples. Given the substantial heterogeneity in effect sizes, identifying when, for whom and under what conditions implementation intentions are most effective is essential. We found the most robust evidence for the sample‐related moderator mean age: Interventions were more effective in studies with younger children. Furthermore, when excluding influential cases, interventions showed stronger effects in clinical samples, which consisted almost exclusively of children with ADHD. These findings suggest that implementation intentions are particularly beneficial for children with self‐regulatory difficulties, whether because they have not yet reached certain developmental milestones or because they have a neurodevelopmental impairment. This aligns with previous research showing particularly strong effects in populations with impaired self‐regulation (e.g. Lengfelder & Gollwitzer, 2001). Notably, we found studies demonstrating strong effects in children as young as 4–5 years old (Mischel & Patterson, 1976; Patterson & Mischel, 1975, 1976). These findings support the idea that implementation intentions are a ‘simple’ strategy (Gollwitzer, 1999) that is especially suitable for children and capable of compensating for developmental or clinical self‐regulation deficits.

For healthy children with well‐developed self‐regulatory capacities, implementation intention effects are only to be expected if the task is difficult enough to demand self‐regulation (Gollwitzer & Brandstätter, 1997; Wieber et al., 2010). Many lab‐based studies used self‐developed tasks, the difficulty level of which was unclear (e.g. Gawrilow et al., 2011b; Gawrilow & Gollwitzer, 2008; Mau et al., 2019; Paul et al., 2007). The fact that several of these studies failed to find significant effects in typically developing children (Gawrilow & Gollwitzer, 2008; Mau et al., 2019; Paul et al., 2007) may indicate ceiling effects. Similar issues may arise in field studies. For instance, Higgins and Conner (2003) aimed to reduce smoking initiation in adolescents, but only three out of 53 adolescents in the control group began smoking, suggesting that the goal was too easy to be influenced by the intervention. Thus, implementation intentions might not be less effective in healthy children if the task is sufficiently challenging.

#### Challenges in testing moderators due to limited variability

##### Convergence on external cues and goal intention control conditions

For many of the additional moderators, limited variability across studies hindered the detection of meaningful effects (see Appendix B). For example, we hypothesized that implementation intentions based on external cues (e.g. ‘if someone offers me a cigarette’) would be more effective than those based on internal cues (e.g. ‘if I feel stressed’), which require introspection and are often less specific or salient. However, only one study used an internal cue (Mau et al., 2019, Study 2), thus preventing any conclusions about this comparison. This lack of variation likely reflects a shared understanding in the literature—grounded in theoretical work by Gollwitzer (1999)—that cues should be concrete and salient (see also Schaaf et al., 2025). Another factor with limited variability was the type of control group. We expected larger effects when implementation intentions were compared to passive control groups (e.g. waitlist or no‐treatment), but only six studies used such designs. Most studies used a goal intention control group, which is standard in implementation intention research. A few studies used multiple control conditions. We found no meaningful differences between these groups.

##### Great variability in materials and instructions for planning

One important set of candidate moderators relates to the question of how to design interventions that optimally support children in formulating effective plans themselves. Only 14 studies involved children generating their own plans; in the remaining 38 studies, plans or examples of plans were provided by the experimenter. Descriptively, interventions with experimenter‐provided plans were more effective. This variable emerged as relevant in the metaforest analysis (after excluding influential cases) but did not quite reach significance. Two studies evaluating the quality of self‐created plans revealed that implementation intentions were only effective when the plans were of high quality (Beall, 2011; Schaaf et al., 2025). This suggests that, while implementation intentions are effective when provided to children, it is unclear how best to support children in generating high‐quality plans themselves. We explored moderators that could point to ways of supporting this process, such as experimenter assistance or the use of pre‐structured sheets that guide plan formulation. However, variation on these variables was limited. Pre‐structured sheets emerged as a significant moderator when the two influential studies were excluded, offering tentative evidence that guided support may improve the quality and effectiveness of self‐generated plans.

Across studies, we found a large variability in the materials and instructions used to provide children with plans or help them develop their own plans. According to Implementation Intention Theory, the effectiveness of implementation intentions depends on the strength of the association between the ‘if’ and ‘then’ components of the plan as well as the plan's accessibility (Achtziger & Gollwitzer, 2018; Webb & Sheeran, 2008). Thus, to strengthen the link between the ‘if’ and ‘then’ components, most studies relied on plan rehearsal. Usually, children were asked to repeat their plan three times (e.g. Bleize et al., 2022; Gawrilow et al., 2011a; Gawrilow et al., 2011b; Kretschmer‐Trendowicz et al., 2021; Mau et al., 2019; Zimmermann & Meier, 2010). However, some studies asked the children to repeat the plan only once (e.g. Duckworth et al., 2013; Yang et al., 2023), and others just instructed children to memorize the plan (e.g. Mauduy et al., 2023). In most cases, children had to write down the plan themselves either digitally (e.g. Breitwieser et al., 2023) or on paper (e.g. Beall, 2011; Crăciun & Băban, 2008). However, especially in preschool children, plans were provided orally (Mischel & Patterson, 1976; Patterson & Mischel, 1975, 1976).

In addition, studies used different strategies to ensure the plan's accessibility. Particularly in laboratory settings, some studies let children formulate a goal intention first in order to activate the goal and thus foster plan accessibility (e.g. Gawrilow et al., 2011a; Gawrilow & Gollwitzer, 2008). In field settings, studies tried to enhance the accessibility of the plan by additional materials like stickers (Rozendaal & Figner, 2020), laminated sheets on which to write the implementation intention (Armitage et al., 2020), posters (Schunk et al., 2022; Schwarz & Gawrilow, 2019) or digital reminders (Breitwieser et al., 2023). Finally, in order to foster children's understanding of implementation intentions and their ability to create well‐formulated plans, some studies provided children with elaborated workbooks (Schunk et al., 2022; Schwarz & Gawrilow, 2019) or short explanatory videos (Armitage et al., 2020; Breitwieser et al., 2023).

This great heterogeneity in materials and instructions might in part reflect different study goals. For example, some studies focused on immediate performance in laboratory tasks, while others focused on long‐term goals, such as improving academic performance or oral health. However, it also demonstrates a lack of best practice guidelines. Most studies do not publish their materials, and descriptions of interventions often lack details on how the plans were created or provided. This makes it difficult to determine which materials work best for which children in which contexts.

##### Teaching implementation intentions as a meta‐strategy

An important question concerns the distinction between simply *having a plan* and learning to use the *strategy of planning*. Very few studies (e.g. Schaaf et al., 2025; Schunk et al., 2022) have aimed to promote the use of implementation intentions as a meta‐strategy, rather than providing pre‐formulated plans or asking children to create one‐off plans. This highlights a significant gap in the literature: How can children be taught to use implementation intentions as a flexible form of self‐regulatory control, and what developmental or instructional factors facilitate this process? While even very young children may benefit from *following* implementation intentions, using them strategically likely requires more advanced cognitive skills: monitoring one's own behaviour, identifying the best strategy for a given goal and context and translating that insight into a well‐formed if‐then plan (Gollwitzer & Schaal, 1998). More research is needed on how and when children develop the capacity to use implementation intentions strategically and how interventions can be designed to scaffold this process effectively.

#### The conceptual importance of plan–behaviour‐goal alignment

Contrary to our expectations, some factors, despite sufficient variability, did not emerge as significant moderators. One such factor was the fit between the plan and the measured outcome. Since implementation intentions target specific behaviours in service of superordinate goals, we expected stronger effects when the measured outcome directly reflected the behaviour specified in the then‐component of the plan. This was the case in 35 studies; in others, the outcome measured the superordinate goal (e.g. increased fruit consumption) or was even more distantly related (e.g. improved self‐regulation ability). The high number of studies with a good fit between the outcome and the plan is probably due to the fact that, if a study reported multiple outcomes, we always selected the outcome with the best fit to the plan. Thus, although this variable was not a significant moderator, our review of the studies suggests it remains conceptually important.

Few studies explicitly addressed the causal chain from plan to behaviour to goal attainment^4^ (see Figure 7). This chain can break down in multiple ways. First, the plan–behaviour contingency may fail, for instance, if the situational cue is too vague to facilitate automatic action initiation. Second, the behaviour‐goal contingency may be weak if the specified behaviour has limited impact on the goal. For example, if a child forms the plan ‘If I watch my favorite TV show, I will eat an apple’, but the show airs only once a week, the impact on overall fruit consumption will be minimal, even with perfect plan execution. Similarly, if a child plans to cope with stress by ‘closing their eyes and ignoring the problem’, the behaviour may be ineffective and the goal of reduced stress will not be achieved.

**FIGURE 7:**
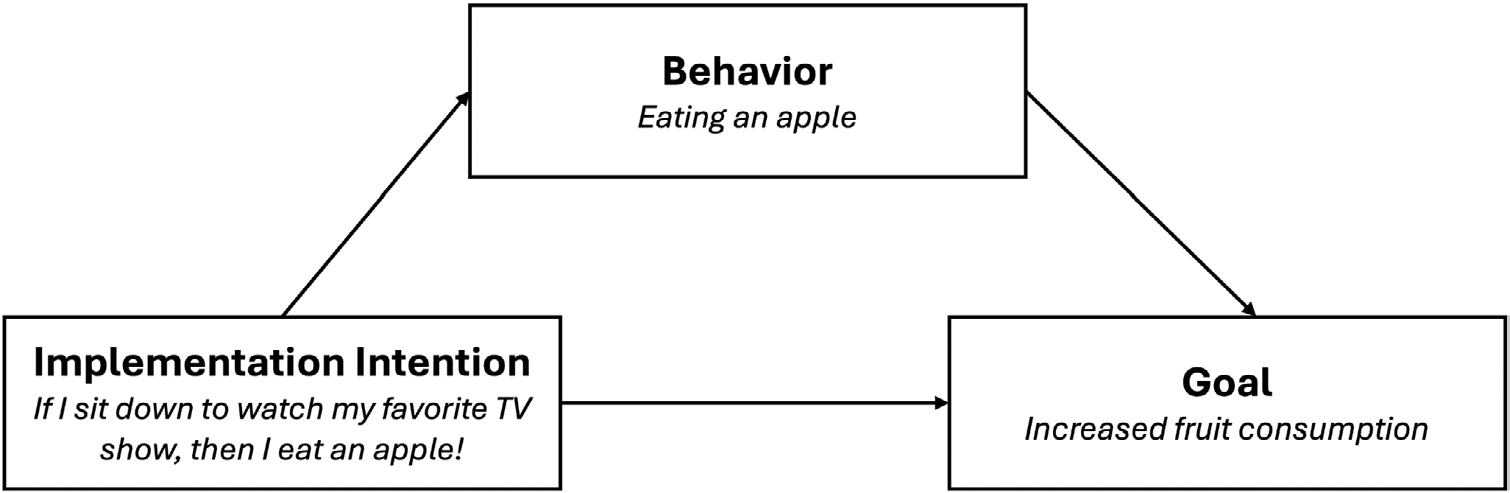
Exemplary causal chain from implementation intentions to goal achievement via behaviour.

Another factor is level‐of‐analysis alignment. That is, the behaviour specified in the plan, the observed behaviour and the measured outcome must be aligned in terms of granularity (Wittmann & Klumb, 2006). For example, Rozendaal and Figner (2020) had children create a plan for using one of four coping strategies against advertising. However, the outcome measure was a composite score reflecting all four coping strategies. This mismatch dilutes the ability to detect whether the specific planned behaviour was enacted. The same logic applies to goal‐level outcomes: if a plan targets a specific academic subskill, but the outcome measure is overall academic achievement, the behaviour‐goal link becomes weak. For instance, Duckworth et al. (2013) instructed children to create a plan for achieving their ‘most important wish or goal that's related to school work’ (p. 748) and used the grade point average (GPA) as the dependent variable. Likewise, Schwarz and Gawrilow (2019) instructed children to plan how to achieve their most important wish for school that could be reached within the next 9 days. The outcome was daily ADHD symptoms. This highlights that the fit between the plan and the outcome is particularly challenging for studies investigating self‐created plans.

In conclusion, even though this factor did not reach statistical significance in our moderator analyses, it is conceptually important that researchers clearly define which effect they aim to assess in the causal chain. This has important implications for study design and interpretation. For example, excluding children who created plans of low quality or could not remember the if‐component of the plan (e.g. Kretschmer‐Trendowicz et al., 2021) may be appropriate if the goal is to examine whether strict treatment fidelity enhances goal attainment. However, such exclusions will bias the results if the aim is to evaluate the full causal chain from forming an implementation intention to behaviour change and, ultimately, goal attainment.

#### Long‐term effectiveness of implementation intentions remains unclear

Another variable that, contrary to our expectations, did not emerge as a significant moderator was the time interval between delivery of the intervention and the first outcome assessment. We had hypothesized that the effects would be stronger when outcomes were measured shortly after the intervention. There was indeed a large range across studies, from same‐day assessments to follow‐ups nearly 4 years later. The absence of a significant decrease in effect sizes for studies with longer time intervals could suggest that implementation intentions may have lasting effects, which is consistent with the idea that they promote instant habit formation (Gollwitzer, 1999). However, this interpretation should be treated with caution. First, the absence of a significant moderating effect cannot be taken as evidence for the absence of moderation. Second, in studies with the longest follow‐up periods, the intervention was typically reinforced through repeated sessions (e.g. Conner et al., 2019; Conner & Higgins, 2010), which likely contributed to the sustained effects. Furthermore, most studies (*n* = 35) assessed outcomes within 24 h of the intervention, which likely limited our ability to detect time effects. Therefore, more research is needed to determine how long the effects of a one‐time implementation intention intervention last, as well as how to prolong them using reminders or booster sessions.

#### Motivation: A critical but underexplored moderator

Beyond the moderators examined at the meta‐analytic level, one particularly important factor that warrants closer attention is motivation. Theoretical and empirical accounts emphasize that implementation intentions are only effective when the individual is committed to the underlying goal (Sheeran et al., 2005). While just over half of the included studies assessed motivation in some form, it was hardly ever tested as a moderator. This is particularly relevant in research with children, where goals are often externally assigned rather than self‐chosen. Unlike adult participants, who typically enrol in studies with pre‐existing personal goals, children may be asked to pursue goals with limited intrinsic relevance. Although a few studies attempted to increase goal value through supplementary materials (e.g. Breitwieser et al., 2023; Conner et al., 2019), the extent to which children were genuinely motivated remains unclear. Four studies combined implementation intentions with mental contrasting (Duckworth et al. 2013; Gawrilow et al., 2013; Schunk et al., 2022; Schwarz & Gawrilow, 2019), a strategy designed to enhance goal commitment when expectations of success are high (Oettingen et al., 2001). While promising, its effectiveness in children and the developmental stage at which it becomes viable remains an open question.

### Objectives and recommendations for future research

There is already substantial evidence demonstrating that implementation intentions can be effective in promoting a wide range of desirable outcomes in children. However, our review of the literature also revealed numerous open questions and shortcomings that, if addressed, could help optimize the design and application of this simple and cost‐effective intervention. Rather than conducting additional studies that merely replicate the general effectiveness of implementation intentions for new goal domains, we encourage researchers to focus on advancing the field conceptually and methodologically. To that end, we conclude this discussion with several concrete recommendations for future research (see Table 2 for an overview). These recommendations are based on an analytical comparison between theory and current research practice, the substantial variability in intervention designs between studies, the moderator analyses and the risk‐of‐bias assessment.

**TABLE 2:** Summary of recommendations.

	Objective	Challenge	Recommendation
(1)	Understand how children form and maintain strong mental representations of plans	Children's cognitive abilities are still developing, likely influencing how they encode and recall plans. Large variety of instructions and materials in current studies.	Explore age‐appropriate formats for representing plans. Test strategies for supporting memory and accessibility (e.g. reminders, visible displays). Develop evidence‐based guidelines for age‐appropriate instructions and materials.
(2)	Ensure children formulate effective plans independently	Children often struggle to create high‐quality plans on their own.	Investigate scaffolds that support effective plan formation. Explore the role of social support, including training for teachers/caregivers.
(3)	Teach if–then planning as a meta‐strategy	The use of if‐then planning as a flexible self‐regulation strategy requires repetition, contextual embedding, scaffolding, and feedback. Developmental prerequisites are not fully understood.	Design interventions that embed planning instruction across contexts and over time. Use scaffolding to support gradual internalization and flexible application of planning. Investigate developmental trajectories to determine when children are ready to benefit from instruction on planning as a meta‐strategy.
(4)	Ensure children are highly committed to both goals and plans	Lack of commitment undermines plan effectiveness.	Ensure equal commitment in intervention and control groups.
(5)	Improve precision and rigour in studying mechanisms of behaviour change	Theoretical assumptions about how implementation intentions influence outcomes are often unspecified. Ambiguities in analytic strategies (e.g. ITT vs. per‐protocol) contribute to bias and inconsistent findings.	Clearly state the proposed mechanism of change. Align outcomes with the targeted level of analysis along the plan–behaviour–goal chain. If broader outcomes are examined, articulate how planning is expected to influence them. Specify analytic approach (intention‐to‐treat vs. per‐protocol) and ensure unbiased implementation.
(6)	Use valid and sensitive outcome measures to detect intervention effects	Implementation intentions are only effective for difficult‐to‐implement goals that demand substantial self‐regulation. Evidence of the sensitivity and validity of outcome measures is often lacking.	Choose tasks and outcomes that reflect goals of appropriate difficulty, which require self‐regulation. Demonstrate psychometric quality of outcome measures.
(7)	Increase transparency in intervention design, analysis, and reporting	Lack of pre‐registration increases the risk of selective reporting or post hoc outcome switching. Variability in intervention instructions/materials reduces comparability and replication.	Pre‐register outcomes and statistical analyses. Share intervention materials openly (e.g. instructions, resources) to reduce variability, enable replication and support creation of optimal materials.
(8)	Strengthen the exchange between research and practice	Practitioners already use plan‐like tools and strategies. Research often overlooks these practical experiences and children's own perspectives.	Draw on practitioner expertise and existing planning practices (medicine, psychotherapy, education). Involve children in co‐designing materials and support measures.

#### Objective 1: Understand how children form and maintain strong mental representations of plans

Due to the ongoing development of cognitive abilities in children, it is essential to understand how cognitive processes influence the effectiveness of planning interventions. For instance, implementation intention theory emphasizes the importance of forming a strong mental representation of the plan in memory and keeping it accessible (Achtziger & Gollwitzer, 2018; Webb & Sheeran, 2008). There is ongoing research in cognitive psychology on how humans represent cognitive concepts, including plans (Kaup et al., 2024). An often‐stated assumption is that representations develop from more modal (i.e. image‐like) to more amodal (i.e. abstract). This suggests that the format in which plans are presented may play a critical role and that the optimal format may vary with age. Visual formats (e.g. drawings) may be more effective than verbal‐only formats for helping children encode and represent the plan in memory. For example, some studies included in this meta‐analysis used narrative videos to explain the concept of implementation intentions (e.g. Armitage et al., 2020; Breitwieser et al., 2023). Other studies have begun exploring child‐friendly ways to present and memorize implementation intentions, using emojis as image‐based representations (Biedermann et al., 2025). Furthermore, research on frequent plan reminders suggests that children may benefit from support in maintaining and recalling their implementation intentions over time (Breitwieser et al., 2023). Strategies such as visibly displaying the plan in the classroom, attaching it to a desk or embedding it into daily routines may enhance accessibility and support plan execution. The large variety of instructions and materials we encountered across studies highlights the need for evidence‐based guidelines on designing age‐appropriate materials that support the cognitive processes involved in planning.

#### Objective 2: Ensure children formulate effective plans independently

While many studies have demonstrated that implementation intentions can be effective when provided to children, children struggle to formulate high‐quality plans on their own (Beall, 2011; Schaaf et al., 2025). Thus, future research should focus on teaching children how to create effective implementation intentions. One promising approach is to use scaffolds, for instance in the form of pre‐structured planning sheets, to guide children through the process of identifying a relevant cue and linking it to a concrete behaviour (e.g. Crăciun & Băban, 2008). Other strategies that are more time‐consuming and resource‐intensive involve extensive workbooks (Schunk et al., 2022). These workbooks are used across multiple lessons in which children learn about goal setting and planning. More research is needed to determine the optimal degree and type of scaffolding.

In addition to structured materials, social support may play a key role in teaching effective plan formulation. Teachers, parents or other caregivers can guide children in identifying meaningful cues and goal‐directed actions. However, providing such social support requires additional training (e.g. 3 h in Schunk et al., 2022), because, in general, interventions delivered by study personnel usually produce larger effects than those delivered by teachers (Beelmann et al., 2014). Given the growing use of digital tools to promote planning skills (Breitwieser et al., 2023; Schaaf et al., 2025), emerging technologies—particularly AI‐driven feedback systems—could be another promising avenue to help children refine their self‐created plans.

#### Objective 3: Teach if–then planning as a meta‐strategy

Building on the previous recommendation, future interventions should aim not only to teach children how to formulate implementation intentions but also to support them in understanding and using implementation intentions as a meta‐strategy (Gollwitzer & Schaal, 1998). This means helping children recognize when planning is needed, how to generate effective if–then plans across different contexts, and how to evaluate and revise those plans. To be effective, strategy instruction should be repeated and embedded across contexts. Scaffolding, such as frequent prompting (e.g. Schaaf et al., 2025), and feedback on their strategy use may help children gradually internalize the core elements of implementation intentions and learn to apply them flexibly. From a developmental perspective, important questions remain about when children acquire the cognitive prerequisites for this type of strategic planning, including the ability to monitor one's own behaviour, reflect on obstacles and select appropriate strategies.

#### Objective 4: Ensure children are highly committed to both goals and plans

When analysing the effects of implementation intentions, it is crucial to ensure that children are committed to both the goal and the plan. This can be achieved by assessing and statistically controlling for both variables. Alternatively, commitment can be manipulated through interventions such as mental contrasting, if expectations of success are high (see Oettingen et al., 2001). Importantly, such manipulations require a manipulation check. In addition, any commitment manipulations must be applied to the control conditions as well if the goal is to investigate the specific effect of implementation intentions. Otherwise, comparing the treatment and control groups would entail the combined effects of planning and commitment.

#### Objective 5: Improve precision and rigour in studying mechanisms of behaviour change

To improve the precision and utility of future research, theoretical assumptions about how implementation intentions are expected to influence the outcome of interest should be clearly stated. This includes specifying whether the focus of the investigation is on the execution of the planned behavioural response, downstream goal achievement or both. It also requires alignment in the level of analysis across the plan–behaviour–goal chain (Figure 7). When the measured outcome is too broad or distal from the specific behaviour targeted in the plan, intervention effects may be obscured. If broader outcomes are of interest—such as academic achievement or well‐being, which are of high practical relevance—it is crucial to clearly articulate the assumed mechanism by which the planning intervention is expected to influence those outcomes.

Clarifying the analytic approach is equally important. Researchers should explicitly state whether they are estimating an intention‐to‐treat effect or a per‐protocol effect and use appropriate methods to ensure unbiased estimates. Many high risk‐of‐bias ratings in this meta‐analysis stemmed from ambiguities in these decisions, such as excluding participants after randomization (which could bias intention‐to‐treat effects) or failing to verify whether child‐created plans met the criteria for a valid implementation intention (which could bias the per‐protocol effect). Greater clarity in theoretical rationale, measurement alignment and analytic strategy will allow for more rigorous testing of implementation intention effects.

#### Objective 6: Use valid and sensitive outcome measures to detect intervention effects

Implementation intentions are only effective for difficult‐to‐implement goals that demand substantial self‐regulation (Gollwitzer & Brandstätter, 1997; Wieber et al., 2010). Therefore, tasks and corresponding outcome measures must be carefully chosen to reflect an appropriate level of challenge. Otherwise, they may not be sensitive to intervention effects, meaning they may not capture changes brought about by the intervention (Naumann et al., 2020).

Beyond sensitivity to intervention effects, researchers should explicitly demonstrate the psychometric soundness, that is, objectivity, reliability and validity, of the instruments they use to detect intervention effects. Notably, approximately half of the studies in our meta‐analysis used self‐developed or adapted outcome measures without providing evidence of their validity. This lack of validation obscures the extent to which the measured outcomes adequately capture the theoretical constructs the interventions aim to influence.

#### Objective 7: Increase transparency in intervention design, analysis and reporting

Across the studies reviewed, there is a clear need for greater transparency in how implementation intention interventions are designed, analysed and reported. Many studies assess multiple outcomes but fail to specify which ones are theoretically driven or for which hypotheses existed in advance. This lack of transparency complicates interpretation and increases the risk of post hoc outcome switching or selective reporting (Nosek et al., 2018). To improve transparency and overall confidence in the findings, we strongly recommend pre‐registering outcomes and statistical analyses. In addition, we encourage researchers to openly share their materials, including both the planning instructions and any additional resources that were used during the intervention. This will reduce the variability in implementation intention instructions, help create optimal materials and allow others to accurately interpret, replicate and build on previous work.

#### Objective 8: Strengthen the exchange between research and practice

Practitioners such as paediatricians, child psychotherapists or teachers use plans on a daily basis, often very similar to implementation intentions. For instance, there is a long history in medicine of written action plans, which have mainly been used in the treatment of asthma bronchiale (Zemek et al., 2008), but also in the treatment of atopic dermatitis (Thandi et al., 2023). Usually, the written action plan specifies various symptoms or symptom severities (similar to an if‐component of an implementation intention) and corresponding actions (similar to the then‐component of an implementation intention) in a stepwise manner.^5^ Similarly, both action and coping planning are regularly used as part of child cognitive behavioural psychotherapy (e.g. Podell et al., 2010). Research should draw on these practical experiences as well as on children's own thoughts (e.g. using participatory research approaches, Kwon et al., 2018) about designing materials and other support measures that could help them create meaningful plans and adhere to them.

### Practical implications

The results of this meta‐analysis suggest that implementation intentions are an effective strategy for promoting self‐regulation in children. Importantly, they appear to be beneficial even for relatively young children and for those with pronounced self‐regulatory difficulties. In light of these findings, a key practical question concerns how the strategy of implementation intentions can be taught to children in practice and in a scalable manner. Practitioners play a crucial role in supporting children in identifying meaningful goals and in formulating effective plans. However, the most effective ways to provide this support remain currently unclear due to the lack of evidence‐based guidelines and the substantial heterogeneity in materials and procedures used across studies. A good starting point are the efforts by Schunk et al. (2022) and Schaaf et al. (2025) that present classroom‐based workbooks and a child‐friendly mobile application, respectively.

## LIMITATIONS

The substantial variability in intervention protocols, materials, study settings and outcome types across the included studies raises the question of comparability. For instance, a study in which children are given an implementation intention to avoid pressing a button during a computerized Go/No‐Go task in a laboratory setting (Gawrilow & Gollwitzer, 2008) differs greatly from a study in which children learn about implementation intentions and mental contrasting over the course of several weeks at school (Schunk et al., 2022). Nevertheless, in accordance with prior meta‐analyses (Gollwitzer & Sheeran, 2006; Sheeran et al., 2024), we synthesized the evidence across these studies, as implementation intentions are considered a domain‐general self‐regulation strategy applicable to a wide range of goals. However, it is important to consider this variability when interpreting the results.

One way to deal with the heterogeneity across studies is to identify relevant moderators that can explain between‐study differences. Although this meta‐analysis did identify substantial between‐study heterogeneity, our ability to explain this variability was limited. Many of the candidate moderators we pre‐registered did not vary as much between studies as we had expected, thus reducing the power to statistically detect their influence. Furthermore, differences in instructions and materials, as mentioned above, are difficult to capture, especially since many studies do not report the procedure with sufficient detail.

We have emphasized the broad scope of studies included in this meta‐analysis, contributing to high between‐study heterogeneity. At the same time, it could be argued that there are important goals and life domains that are currently underrepresented in the literature, thus limiting the generalizability of our findings. Most studies examined the effect of implementation intentions on executive functions, as tested with computerized tasks. Additional work has been done in the areas of health‐related behaviours (e.g. smoking prevention, oral hygiene) and, to a lesser extent, educational goals. Only two studies in this meta‐analysis targeted interpersonal challenges (Bleize et al., 2022; Mauduy et al., 2023), although peer relations, changes in family dynamics and social identity become increasingly important during early adolescence (Steinberg & Morris, 2001). Many other important areas in children's lives also remain underexplored. For example, research finds high levels of problematic screen time and social media use in children and young adolescents (Nagata et al., 2025), yet none of the studies in this meta‐analysis addressed this challenge. Future research should focus on goals and life domains that are intrinsically motivating yet difficult for children to navigate.

## CONCLUSION

The present meta‐analysis provides robust evidence that implementation intentions are an effective self‐regulation intervention for children across many goal domains and settings. At the same time, the substantial heterogeneity in effect sizes highlights the need for a better understanding of the conditions under which implementation intentions are most effective and for whom. Our findings revealed stronger effects in studies with younger children and children with ADHD. However, our ability to detect the influence of other potentially relevant moderators was limited because many of them had low variability across studies. We offer concrete recommendations to advance the field. First, more systematic research is needed on how children form strong mental representations of implementation intentions, and how they can be supported in creating their own plans and in using if–then planning as a meta‐strategy. Second, we call for greater conceptual clarity in the design and evaluation of implementation intention interventions. Third, we emphasize the need for greater methodological rigour, including the adoption of open science practices and attention to the validity and instructional sensitivity of outcome measures.

## AUTHOR CONTRIBUTIONS

**Jasmin Breitwieser:** Conceptualization; formal analysis; writing – original draft; writing – review and editing; project administration; methodology; investigation. **Tilman Reinelt:** Conceptualization; writing – original draft; writing – review and editing; methodology; investigation.

## FUNDING INFORMATION

We received no specific funding for this work.

## CONFLICT OF INTEREST STATEMENT

The authors declare no conflicts of interest.

## Data Availability

The data that support the findings of this study are openly available on the Open Science Framework at https://doi.org/10.17605/OSF.IO/YD623.

## Footnotes

^1^
Example search string used in PsycInfo. SU: subject; AG: age group. All search strings are displayed in Supplement S3 on the OSF project page.

^2^
At the time of conducting the search, the study by Schaaf et al. (2025) was still an unpublished manuscript (preprint). The results were coded based on the preprint; they did not change in the published version.

^3^
Two effect sizes reported by Toli et al. (2016) for Gawrilow et al. (2011a), Study 2, were not considered here. These were extreme outliers (*d* = 3.99 and *d* = 4.99) and were also excluded by Toli et al. from their main analyses. We were unable to reproduce these values; based on our coding, the effect size for this study is *g* = 0.96.

^4^
Figure 7 also contains a direct pathway from the plan to the desired goal, because research suggests that implementation intentions can affect goal attainment not only through execution of the intended behaviour but also through other mechanisms, some of which may even be detrimental. For example, studies show that plans may prevent individuals from seizing alternative opportunities for goal‐directed behaviour not covered by the plan (Masicampo & Baumeister, 2012).

^5^
Since medical studies compare written action plans to standard care, which likely includes action plan formulation without the written component, these studies were excluded from the present meta‐analysis.

## APPENDIX A Studies included in the meta‐analysis

### A.1

**Table:**

Study	Country^a^	Sample				Intervention	Outcome
		*N*	*M*_Age_ (*SD*)	% female	Clinical?		
Between‐subject							
Araújo‐Soares et al. (2009)	PRT	291	12.13 (1.01)			The intervention group participated in an intervention program that included 12 weekly 90‐min sessions focused on physical activity and other health‐related goals. One session focused on formulating specific action plans for when, where, and how to be physically active, and coping plans for dealing with barriers. The control group received no intervention.	Self‐reported physical activity in minutes per week
Armitage and Sprigg (2010)	GBR	77	8.06 (1.63)	52.58%		All groups were ascribed the goal to run around as much as possible. The intervention group said the plan out loud three times: ‘If it is playtime, then I will run around as much as possible’. The control group said the goal out loud three times.	Self‐reported physical activity
Armitage et al. (2020)	GBR	42	7.63 (1.55)	49.35%	Repaired cleft lip and/or palate	All participants received standard dental care information. In the two intervention groups, parents and children together formed implementation intentions to improve oral hygiene behaviour. In one of the intervention groups, adults received a reminder of the implementation intention after 3 months. The control group received the same materials but did not form plans.	Dental plaque presence Gingival inflammation
Beall (2011)	USA	160	NR	44.83%		All participants received magazines that they were asked to read. Two implementation intention groups formed plans for when and where to read, and for overcoming obstacles to reading the magazines. The groups only differed in whether the students could choose which of the two target articles to read. The two control groups formulated no plans.	Amount of reading completed
Bleize et al. (2022)	NLD	268	12.99 (0.84)	53.14%		The three intervention groups received different behaviour change interventions to reduce conformity to cyber aggression. They formed implementation intentions about what to do when people in their messaging group acted aggressive towards people outside of the group. The control group completed questions and tasks about cyberbullying.	Conformity to cyber aggressive attitudes Conformity to cyber aggressive intentions
Boscher (2006), Study a	DEU		9.32 (0.97)	6.45%	ADHD	All participants performed a modified Number Stroop Task and formed the goal intention ‘I count the numbers!’. Intervention Group 1 formed a task‐continuing plan (‘And whenever a new number series appears, I concentrate on the number set!’). Intervention Group 2 formed a temptation‐inhibiting plan (‘And whenever a new number line appears, I ignore the number type!’). The control group formed no plan.	Reaction times in a modified Number Stroop Task Number of errors
Boscher (2006), Study b	DEU		8.82 (1.01)	46.88%		Same as Boscher (2006), Study a, but with a non‐clinical sample.	Reaction times in a modified Number Stroop Task Number of errors
Breitwieser et al. (2023)	DEU; AUT	130	10.75 (0.43)	46.1%		All participants watched a video about the benefits of distributed practice with a vocabulary app. The two intervention groups formed an implementation intention about when and where to use the app over the next 36 days. One of these groups received reminders of the plan. The control group received reminders about the benefits of distributed practice.	Frequency of studying with vocabulary app
Conner and Higgins (2010)	GBR	1338	11.5 (NR)^b^	47.69%		All participants read information about the effects of smoking. They then committed to not smoking. In the intervention group, participants read antismoking messages, formed an implementation intention about what to say when someone offered them a cigarette, and indicated where they would not smoke. Control Group 1 received a self‐efficacy intervention. Control Group 2 completed an implementation intention for an unrelated behaviour. Control Group 3 completed a series of self‐efficacy statements about how they might complete all their schoolwork. The intervention protocol was repeated at 4, 8, 12, 16, 20 and 24 months post baseline.	Self‐reported smoking behaviour Breath carbon monoxide (CO)
Conner et al. (2019)	GBR	5974	11.5 (NR)^b^	50.07%		The intervention group read antismoking messages and formed an implementation intention about what to say when someone offered them a cigarette. They indicated where they would not smoke. The control group read pro‐homework messages and formed an implementation intention on that topic. The intervention protocol was repeated 12‐, 24‐, 36‐, and 48‐months post baseline.	Self‐reported ever smoking Breath carbon monoxide (CO)
Crăciun and Băban (2008)	ROU	233	10.06 (0.25)	50.43%		The intervention group formed an implementation intention specifying when, where and how to eat a fruit snack during school breaks over the next 2 weeks. The control group received no additional instructions.	Self‐rated fruit snack intake
Duckworth et al. (2013)	USA	77	11.05 (0.69)	51.07%		All participants selected a goal related to schoolwork and wrote down the best outcome of achieving the goal. The intervention group performed mental contrasting with implementation intentions (MCII), forming a plan about how to overcome an obstacle that stands in the way of achieving the goal. The positive‐thinking control group wrote down another good outcome of achieving the goal and thought about its attainment.	Grade Point Average (GPA) Conduct (teacher‐rated)
Gawrilow and Gollwitzer (2008), Study 1a	DEU	30	10.94 (1.43)	52%	ADHD	All participants set the goal to not press any key when a sound occurred together with the stimulus. The intervention group formed an implementation intention to stop correctly on No‐Go trials (‘And if I hear a tone, then I will not press any key!’). The control group formed no plan.	Percentage correct of all No‐Go trials
Gawrilow and Gollwitzer (2008), Study 1b	DEU	28	11.53 (1.1)	0%		Same as Gawrilow and Gollwitzer (2008), Study 1a, but with a non‐clinical sample.	Percentage correct of all No‐Go trials
Gawrilow and Gollwitzer (2008), Study 2	DEU	20	11.15 (1.65)	0%	ADHD	Same as Gawrilow and Gollwitzer (2008), Study 1a, but participants completed the task twice (first without medication, second with medication).	Percentage correct of all No‐Go trials
Gawrilow et al. (2011a), Study 1a	DEU	43	9.58 (1.23)	0%	ADHD	All participants performed an adapted Wisconsin Card Sorting task. The intervention group set themselves the goal to match as many cards as possible (‘I will match as many cards as possible according to the rule!’) and formed the implementation intention ‘Whenever my rule is wrong, then I will try another rule!’. Control Group 1 memorized a sentence summarizing the task instructions, Control Group 2 received no additional instruction.	Percentage of perseverative errors
Gawrilow et al. (2011a), Study 1b	DEU	36	8.59 (0.47)	0%		Same as Gawrilow et al. (2011a), Study 1a, but with a non‐clinical sample.	Percentage of perseverative errors
Gawrilow et al. (2011a), Study 2a	DEU	33	10.33 (1.12)	0%	ADHD	All participants were assigned the goal of not being distracted during a math task (‘I won't get distracted!’). The two intervention groups formed an implementation intention to help them stay focused (Group 1: ‘Whenever the movie starts, then I will work hard on my task!’; Group 2: ‘Whenever the movie starts, then I will ignore it!’). The control group received no additional instruction.	Time to solve math problems
Gawrilow et al. (2011a), Study 2b	DEU	47	10.73 (1.19)	0%		Same as Gawrilow et al. (2011a), Study 2a, but with a non‐clinical sample.	Time to solve math problems
Gawrilow et al. (2011b), Study 1	DEU	45	10.73 (1.39)	0%	ADHD	All participants performed a delay of gratification task. The intervention group formed an implementation intention related to maximizing points in the task (‘Whenever a red picture appears, then I will wait for the blue one’). Control Group 1 repeated a sentence summarizing task instructions; Control Group 2 formed a goal intention.	Points earned in delay of gratification task
Gawrilow et al. (2011b), Study 2a	DEU	47	10.34 (1.59)	31.11%	ADHD	Similar to Gawrilow et al. (2011b), Study 1, with slight modifications to the delay time in the task.	Points earned in delay of gratification task
Gawrilow et al. (2011b), Study 2b	DEU	40	11.27 (1.44)	0%		Same as Gawrilow et al. (2011b), Study 2a, but with a non‐clinical sample.	Points earned in delay of gratification task
Gawrilow et al. (2013)	DEU	116	12.57 (0.89)	0%		All participants completed a learning style intervention. In addition, the intervention group performed mental contrasting with implementation intentions (MCII) to improve management of school‐related activities.	Management of school‐related activities (parent‐reported)
Gholami et al. (2015)	IRA	166	12.5 (1.14)	32.76%		The intervention group received an intervention package and formed action and coping plans to promote regular dental flossing. For action planning, participants were asked to generate plans for two occasions by specifying the place and the time that they wanted to floss their teeth. For coping planning, participants were asked to generate two situations which may impede the planned behaviour and a strategy to overcome the barriers. The control group received no intervention (waitlist control group).	Frequency of dental flossing (self‐reported)
Higgins and Conner (2003)	GBR	162	11.5^b^	100%		All participants read information about the health consequences of smoking. The intervention group formed an implementation intention about what to say when someone offered them a cigarette. They indicated where they would not smoke. The control group formed an implementation intention related to an unrelated behaviour.	Smoking behaviour (self‐reported)
Kretschmer‐Trendowicz et al. (2021), Study 1a	DEU	57	9.1 (0.31)	49.49%		All participants performed a prospective memory task. The intervention group formed implementation intentions to support prospective memory performance (‘When I see RLRLR on the screen, then I will press the pink button’). The control group was asked to say what they had to do when they saw the cues on the screen.	Prospective memory accuracy Prospective memory reaction times
Kretschmer‐Trendowicz et al. (2021), Study 1b	DEU	57	12.17 (0.48)	66.67%		Same as Kretschmer‐Trendowicz et al. (2021), Study 1a, but with school‐aged children.	Prospective memory accuracy Prospective memory reaction times
Kretschmer‐Trendowicz et al. (2021), Study 2a	DEU	37	9 (0.33)	53.19%		Similar to Kretschmer‐Trendowicz et al. (2021), Study 1a, with higher cognitive demand of the ongoing task in the prospective memory paradigm.	Prospective memory accuracy Prospective memory reaction times
Kretschmer‐Trendowicz et al. (2021), Study 2b	DEU	46	12^b^	59.46%		Same as Kretschmer‐Trendowicz et al. (2021), Study 2a, but with school‐aged children.	Prospective memory accuracy Prospective memory reaction times
Mau et al. (2019), Study 1	DEU	151	7.3 (0.9)	43.48%		Similar to Gawrilow et al. (2011b). At the end, participants could exchange the points earned in the delay of gratification task into candy.	Points earned in delay of gratification task
Mau et al. (2019), Study 2	DEU	134	6.4 (0.5)	55%		All participants performed a delay of gratification task in the context of buying items at a kiosk. The intervention group formed an implementation intention to help avoid the temptation to buy immediately (‘As soon as I want to buy something for 30 cents, I'll remember the bigger reward, and wait.’). Control Group 1 received no additional instruction; Control Group 2 repeated the rule of the game.	Whether or not a child bought at least one snack during the delay‐of‐gratification task
Mauduy et al. (2023)	FRA	101	12.7 (0.77)	61%		The intervention group collectively discussed school bullying situations and formed three implementation intentions to increase victim‐defending behaviour in bullying situations. Control Group 1 collectively reflected on their past bullying behaviour and developed arguments against bullying; Control Group 2 received no additional instruction.	Victim‐defending behaviours (self‐reported)
Mischel and Patterson (1976)	USA	40	4.5 (NR)	50%		All participants promised both to work hard and to resist Mr. Clown Box, should he tempt the child to stop working on a pegboard task. Intervention Group 1 received an inhibition plan (‘So that's what you can do. He says, “Look,” and you say, “No, I'm not going to look at Mr. Clown Box.”’). Intervention Group 2 received a reward‐oriented plan (‘So that's what you can do. He says, “Look,” and you say, “I want to play with the fun toys and Mr. Clown Box later.”’), Intervention group 3 received a task‐facilitating plan (‘So that's what you can do. He says, “Look,” and you say, “I'm going to look at my work.”‘). Control Group 1 received an irrelevant plan and Control Group 2 received no plan.	Number of separate departures from working on the task in a resistance to temptation paradigm (Mr. Clown Box Paradigm) Mean length of each departure from the task
Patterson and Mischel (1975), Study 1	USA	35	4.75 (NR)	48.57%		All participants promised both to work hard and to resist Mr. Clown Box, should he tempt the child to stop working on a copying task. Intervention Group 1 received a plan (‘So that's what you can do. He says, “Look,” and you say, “No, I can't; I'm working.” And when you say it, do it.’). Intervention Group 2 received the same plan and in addition rehearsed the plan. Control Group 1 played irrelevant verbal and cognitive games whereas Control Group 2 received no plan.	Amount of time children spent working in a Mr. Clown Box Paradigm
Patterson and Mischel (1975), Study 2	USA	36	4.5 (NR)	47.22%		Same instructions as in Study 1 but Intervention Group 1 received three plans and Intervention Group 2 received one plan. The control group received no plans.	Amount of time children spent working in a Mr. Clown Box Paradigm
Patterson and Mischel (1976)	USA	48	4.75 (NR)	16.67%		Instructions for all participants were intended to ensure that all participants were motivated to complete the task and to resist the temptation. Intervention Group 1 received a task‐facilitating plan (‘When Mr. Clown Box says to look at him and play with him, then you can just look at the pegboard and say “I'm going to look at my work.”‘). Intervention Group 2 received a temptation‐inhibiting plan (‘When Mr. Clown Box says to look at him and play with him, then you can just not look at him, and say “I'm not going to look at Mr. Clown Box.”‘). Intervention Group 3 received a combination plan (‘When Mr. Clown Box says to look at him and play with him, then you can just look at the pegboard, not at him, and say “I'm not going to look at Mr. Clown Box; I'm going to look at my work.”‘). The control group received no plans.	Proportion of time spent working in a Mr. Clown Box Paradigm Mean length of each distraction
Rozendaal and Figner (2020)	NLD	704	9.22 (0.92)	48%		The intervention group participated in a program consisting of six 60‐min sessions that focused on advertising literacy and coping strategies. They were instructed to choose their most preferred coping strategy and complete a pre‐printed if‐then plan in a pre‐printed format (‘If I see an ad, then I will [coping strategy]’). The control group did not participate in the program and received no plans.	Use of advertising coping strategies
Schneikert (2005)	DEU	43	9.58 (1.23)	0%	ADHD	Children in the intervention group received a goal intention ‘For the card game to work well, please remember the following: Your goal for the card game is: “I want to match as many cards as possible according to the correct rule!” and the following plan: “If my rule is wrong, I'll try another rule straight away!” Children in Control Group 1 received nothing, children in Control group 2 received the same goal intention but without the plan.	Number of perseverative errors in a Wisconsin Card Sorting Test Percentage of perseverative errors out of total number of errors
Schunk et al. (2022)	DEU	572	6.84 (0.36)	53.78%		The intervention group received five scripted school lessons. The participants learned about mental contrasting with implementation intentions (MCII) and applied it to three goals, including a reading practice goal. The control group received regular classroom teaching.	Reading comprehensions skills
Schwantje (2005), Study a	DEU	32	10.28 (1.09)	0%	ADHD	Both intervention groups first received a goal intention. Intervention Group 1 additionally received task‐related implementation intentions (‘Whenever I see moving pictures, then I continue to calculate with particular concentration!’ and ‘Whenever I hear sounds, then I continue to calculate with particular concentration!’). Intervention Group 2 additionally received distraction‐related implementation intentions (‘Whenever I see moving images, then I ignore them completely!’ and ‘Whenever I hear sounds, then I ignore them completely!’). The control group only received the goal intention.	Number of math tasks completed during periods of distraction Mean response times during periods of distraction
Schwantje (2005), Study b	DEU	41	10.70 (1.20)	0%		Same as Schwantje (2005), Study a, but with a non‐clinical sample.	Number of math tasks completed during periods of distraction Mean response times during periods of distraction
Schwarz and Gawrilow (2019)	DEU	47	11.2 (0.7)	51.05%		All participants formulated a goal relevant to what they wanted to accomplish in school and imagined the positive state of having accomplished that goal. The intervention group then identified an obstacle and formulated an implementation intention to overcome it (MCII). The control group formed an if‐then statement related to the feeling of achieving the goal.	ADHD symptoms (parent‐reported)
Simpriano and Mialhe (2017)	BRA	160	8.5^b^	61.22%		All participants received an educational intervention focused on teaching children about oral health through a variety of interactive activities and resources. The two intervention groups formed an implementation intention specifying when, where and how to brush their teeth. In addition, children were oriented to think of situations that would prevent them from performing the behaviour and the best way to overcome them. In Intervention Group 2, parents attended a lecture and were recommended to monitor the children.	Plaque Index Gingival Bleeding Index
Stumpf (2004), Study a	DEU	42	10.45 (1.63)	0%	ADHD	All participants performed the Six Part Test. Children in the intervention group were asked to remember the following sentences: ‘Whenever I have completed five tasks in a box, I switch to another box.’ and ‘Whenever I have done one color, I switch to another color.’ Control Group 1 received a goal intention whereas Control Group 2 just had to repeat the task instructions.	Performance (number of points) in the Six Part Test
Stumpf (2004), Study b	DEU	47	11.45 (1.46)	0%		Same as Stumpf (2004), Study a, but with a non‐clinical sample.	Performance (number of points) in the Six Part Test
Thompson et al. (2015)	USA	387	10^b^	NR		All participants played a 10‐episode online video game aimed at promoting healthy eating. Intervention Group 1 created an action plan specifying how (i.e. who, what, when) they would meet the goal. Intervention Group 2 created a coping plan that identified a potential barrier that might keep them from meeting their goal. Intervention Group 3 created both the action and the coping plan. The control group played the game without setting goals or receiving plans.	Combined fruit and vegetable intake (self‐reported via 24 h dietary recall over the phone)
Wieber et al. (2011)	DEU	49	6.75 (0.75)	52.7%		All participants performed a categorization task involving distractors. The intervention group formed an implementation intention ‘If there is a distraction, then I will ignore it!’. The control group stated a shielding goal intention (‘I will ignore distractors’).	Response time differences (critical trials minus baseline trials) Response time differences (critical trials minus control trials)
Yang et al. (2023)	CHN	154	9.04 (1.43)	53.06%		All participants performed a prospective memory task. The intervention group formed an implementation intention to improve prospective memory performance (‘Whenever I see an animal word I will press the 1‐key with my left index finger as quickly as possible’). The control group received the prospective memory instructions and a confirmation question that they understood the task.	Accuracy scores of time‐based prospective memory
Zimmermann and Meier (2010)	CHE	92	12.8 (1.1)	49.35%		All participants performed a prospective memory task. The intervention group formed an implementation intention (‘Whenever I see an animal word I will press the 1‐key with my left index finger as quickly as possible’). The control group only received the prospective memory instructions.	Proportion of correct responses
Within‐subject							
Paul et al. (2007), Study a	DEU	13	12.4 (0.37)	47.5%	ADHD	Participants performed a Go/No‐Go task, initially without an implementation intention. In a second session, they formed the implementation intention ‘If I see a hand, then I will not press any button.’	Number of correctly inhibited responses following No‐Go trials
Paul et al. (2007), Study b	DEU	16	12.9 (0.33)	7.69%		Same as Paul et al. (2007), Study a, but with a non‐clinical sample.	Number of correctly inhibited responses following No‐Go trials
Schaaf et al. (2025)	DEU	357	11.63 (0.69)	6.25%		The participants used a vocabulary learning app as frequently as possible over 42 days. On some days, they received planning prompts, asking them to form an implementation intention, specifying when and where to study the next day (intervention condition). On other days, they received no such prompt (control condition).	Whether or not a child learned at least one word in the vocabulary app (on any given day)

*Notes*: The values presented here are based on the reported age range; NR = Not reported.

*Abbreviation*: NR, not reported.

^a^ Mean age was not reported.

^b^ Country of data collection (codes according to ISO 3166‐1 alpha‐3).

## APPENDIX B Overview of moderators

### B.1

**Table:**

Moderator	Variable type	Number of effect sizes	*M* (SD)	Association with effect Size^a^			
				Estimate	SE	95% CI	*p*
Population							
Type of sample	*Categorical* Non‐clinical (partly) clinical sample	40 12		−0.29	0.14	[−0.55, −0.02]	.036
Mean age	*Continuous*	51	9.93 (2.29)	−0.06	0.02	[−0.11, −0.02]	.009
Gender	*Continuous* %female	50	33.92 (25.85)	0.00	0.00	[−0.00, 0.00]	.960
Intervention protocol							
Number of different plans each participant received/created	*Categorical* One plan More than one plan	32 19		−0.24	0.10	[−0.44, −0.03]	.022
Plan creation	*Categorical* Self‐created Selection from provided examples experimenter‐provided	14 5 33		−0.06 0.16	0.16 0.11	[−0.38, 0.26] [−0.06, 0.38]	.702 .155
Experimenter assistance with plan creation	*Categorical* No assisted planning Assisted planning	10 2		−0.10	0.23	[−0.55, 0.34]	.648
Provision of cue‐component	*Categorical* Derived from pilot data Other	1 38		0.05	0.41	[−0.76, 0.86]	.901
Provision of then‐component	*Categorical* Pilot data Other	1 40		0.04	0.39	[−0.72, 0.79]	.927
Setting	*Categorical* Field Laboratory	20 32		0.15	0.10	[−0.05, 0.35]	.141
Time between intervention and first outcome assessment	*Continuous*	52	67.04 (283.11)	−0.00	0.00	[−0.00, 0.00]	.449
Fit of outcome with plan	*Categorical* Outcome is correlate of intended goal Outcome reflects intended goal Outcome directly relates to then‐component of the plan	3 14 35		0.11 0.14	0.23 0.22	[−0.34, 0.55] [−0.28, 0.56]	0.646 0.519
Pre‐registration of outcomes	*Categorical* No pre‐registration Pre‐registered	46 6		−0.16	0.14	[−0.43, 0.12]	.262
Action versus coping plan	*Categorical* Action plan Coping plan	27 18		0.09	0.13	[−0.17, 0.34]	.515
Type of if‐component	*Categorical* Internal External	1 45		−0.26	0.40	[−1.04, 0.52]	.513
Type of then‐component	*Categorical* Internal External	9 32		−0.19	0.17	[−0.52, 0.14]	.257
Format of the plan	*Categorical* If‐then If‐then‐why Prestructured sheet Free format	47 0 4 0		0.21	0.17	[−0.12, 0.55]	.215
Number of people involved in planning	*Categorical* Individual Cooperative	49 3		−0.14	0.20	[−0.53, 0.25]	.474
Type of control group	*Categorical* Same intervention with different content (i.e. if‐then plan on a different goal) Different intervention (e.g. self‐efficacy training) Similar procedure without if‐then plan (e.g. same goal intention and materials, but no plan) Nothing (e.g. wait list control group) Mixed: multiple control groups without relevant goal setting	Categorical 2 2 29 6 11		0.12 0.14 0.02 0.17	0.39 0.26 0.29 0.29	[−0.64, 0.88] [−0.38, 0.66] [−0.54, 0.59] [−0.39, 0.73]	.755 .592 .942 .545

*Note*. Moderators highlighted in grey were not part of the main metaforest analysis as they only apply to a subsample of studies.

^a^ Results from a meta‐regression with a single predictor.

## APPENDIX C Effect size by publication year

### C.1

This figure was created with the ‘ggplot2’ package, version 3.5.2 (Wickham, 2016).

## References

[bjop70065-bib-0001] Achtziger, A. , Bayer, U. C. , & Gollwitzer, P. M. (2012). Committing to implementation intentions: Attention and memory effects for selected situational cues. Motivation and Emotion, 36(3), 287–300. 10.1007/s11031-011-9261-6

[bjop70065-bib-0002] Achtziger, A. , & Gollwitzer, P. M. (2018). Motivation and volition in the course of action. In J. Heckhausen & H. Heckhausen (Eds.), Motivation and action (pp. 485–527). Springer. 10.1007/978-3-319-65094-4_12

[bjop70065-bib-0003] Achtziger, A. , Gollwitzer, P. M. , & Sheeran, P. (2008). Implementation intentions and shielding goal striving from unwanted thoughts and feelings. Personality and Social Psychology Bulletin, 34(3), 381–393. 10.1177/0146167207311201 18272806

[bjop70065-bib-0004] Adriaanse, M. A. , de Ridr, D. T. D. , & Wit, J. B. F. (2009). Finding the critical cue: Implementation intentions to change one's diet work best when tailored to personally relevant reasons for unhealthy eating. Personality and Social Psychology Bulletin, 35(1), 60–71. 10.1177/0146167208325612 19106078

[bjop70065-bib-0005] Adriaanse, M. A. , Vinkers, C. D. W. , de Ridr, D. T. D. , Hox, J. J. , & de Wit, J. B. F. (2011). Do implementation intentions help to eat a healthy diet? A systematic review and meta‐analysis of the empirical evidence. Appetite, 56(1), 183–193. 10.1016/j.appet.2010.10.012 21056605

[bjop70065-bib-0006] * Araújo‐Soares, V. , McIntyre, T. , MacLennan, G. , & Sniehotta, F. F. (2009). Development and exploratory cluster‐randomised opportunistic trial of a theory‐based intervention to enhance physical activity among adolescents. Psychology & Health, 24(7), 805–822. 10.1080/08870440802040707 20205028

[bjop70065-bib-0007] * Armitage, C. J. , & Sprigg, C. A. (2010). The roles of behavioral and implementation intentions in changing physical activity in young children with low socioeconomic status. Journal of Sport & Exercise Psychology, 32(3), 359–376. 10.1123/jsep.32.3.359 20587823

[bjop70065-bib-0008] * Armitage, C. J. , Walsh, T. , Mooney, J. , Tierney, S. , & Callery, P. (2020). Proof of concept trial for a new theory‐based intervention to promote child and adult behavior change. Journal of Behavioral Medicine, 43(1), 80–87. 10.1007/s10865-019-00061-0 31154601

[bjop70065-bib-0009] Atance, C. M. , & Metcalf, J. L. (2013). Future thinking in young children. In M. Taylor (Ed.), The Oxford handbook of the development of imagination (pp. 305–324). Oxford University Press.

[bjop70065-bib-0010] Balduzzi, S. , Rücker, G. , & Schwarzer, G. (2019). How to perform a meta‐analysis with R: A practical tutorial. Evidence‐Based Mental Health, 22(4), 153–160. 10.1136/ebmental-2019-300117 PMC10231495 31563865

[bjop70065-bib-0011] Bartoš, F. , & Schimmack, U. (2022). Z‐curve 2.0: Estimating replication rates and discovery rates. Meta‐Psychology, 6, 2720. 10.15626/MP.2021.2720

[bjop70065-bib-0012] Baujat, B. , Mahé, C. , Pignon, J.‐P. , & Hill, C. (2002). A graphical method for exploring heterogeneity in meta‐analyses: Application to a meta‐analysis of 65 trials. Statistics in Medicine, 21(18), 2641–2652. 10.1002/sim.1221 12228882

[bjop70065-bib-0013] Bayer, U. C. , Achtziger, A. , Gollwitzer, P. M. , & Moskowitz, G. B. (2009). Responding to subliminal cues: Do if‐then plans facilitate action preparation and initiation without conscious intent? Social Cognition, 27(2), 183–201. 10.1521/soco.2009.27.2.183

[bjop70065-bib-0014] Beall, L. C. (2011). *Implementation intentions as a means of promoting voluntary reading behavior*. Unpublished Doctoral Dissertation. University of Maryland, Baltimore County.

[bjop70065-bib-0015] Beelmann, A. , Pfost, M. , & Schmitt, C. (2014). Prävention und Gesundheitsförderung bei Kindern und Jugendlichen: Eine Meta‐Analyse der deutschsprachigen Wirksamkeitsforschung [Prevention and health promotion in children and adolescents: A meta‐analysis of German‐language effectiveness research]. Zeitschrift für Gesundheitspsychologie, 22(1), 1–14. 10.1026/0943-8149/a000104

[bjop70065-bib-0016] Begg, C. B. , & Mazumdar, M. (1994). Operating characteristics of a rank correlation test for publication bias. Biometrics, 50(4), 1088–1101. 10.2307/2533446 7786990

[bjop70065-bib-0017] Bélanger‐Gravel, A. , Godin, G. , & Amireault, S. (2013). A meta‐analytic review of the effect of implementation intentions on physical activity. Health Psychology Review, 7(1), 23–54. 10.1080/17437199.2011.560095

[bjop70065-bib-0018] Biedermann, D. , Breitwieser, J. , Nobbe, L. , Drachsler, H. , & Brod, G. (2025). Memorizing plans with an app: Large individual differences in the effectiveness of retrieval‐based and generative learning activities in a naturalistic context. Learning and Individual Differences, 118, 102641. 10.1016/j.lindif.2025.102641

[bjop70065-bib-0019] Bieleke, M. , Keller, L. , & Gollwitzer, P. M. (2021). If‐then planning. European Review of Social Psychology, 32(1), 88–122. 10.1080/10463283.2020.1808936

[bjop70065-bib-0020] * Bleize, D. N. M. , Anschutz, D. J. , Tanis, M. , & Buijzen, M. (2022). Testing a first online intervention to reduce conformity to cyber aggression in messaging apps. PLoS One, 17(8), 1–23. 10.1371/journal.pone.0272615 PMC9362912 35944038

[bjop70065-bib-0022] * Boscher, M.‐L. (2006). *Hemmung automatischer Reaktionen durch Vorsätze bei ADHS‐Kindern: Eine Vergleichsstudie von ADHS‐ und Kontrollkindern* [Inhibition of automatic responses through implementation intentions in children with ADHD: A comparative study of children with ADHD and control children]. Unpublished Diploma Thesis, University of Konstanz, Germany.

[bjop70065-bib-0023] Brandstätter, V. , Lengfelder, A. , & Gollwitzer, P. M. (2001). Implementation intentions and efficient action initiation. Journal of Personality and Social Psychology, 81(5), 946–960. 10.1037//0022-3514.81.5.946 11708569

[bjop70065-bib-0024] * Breitwieser, J. , Nobbe, L. , Biedermann, D. , & Brod, G. (2023). Boosting self‐regulated learning with mobile interventions: Planning and prompting help children maintain a regular study routine. Computers & Education, 205(12), 104879. 10.1016/j.compedu.2023.104879

[bjop70065-bib-0025] Burkard, C. , Rochat, L. , van der Juillerat Linden, A.‐C. , Gold, G. , & van der Linden, M. (2014). Is working memory necessary for implementation intentions to enhance prospective memory in older adults with cognitive problems? Journal of Applied Research in Memory and Cognition, 3(1), 37–43. 10.1016/j.jarmac.2014.01.004

[bjop70065-bib-0026] Carraro, N. , & Gaudreau, P. (2013). Spontaneous and experimentally induced action planning and coping planning for physical activity: A meta‐analysis. Psychology of Sport and Exercise, 14(2), 228–248. 10.1016/j.psychsport.2012.10.004

[bjop70065-bib-0027] Carter, E. C. , Schönbrodt, F. D. , Gervais, W. M. , & Hilgard, J. (2019). Correcting for bias in psychology: A comparison of meta‐analytic methods. Advances in Methods and Practices in Psychological Science, 2(2), 115–144. 10.1177/2515245919847196

[bjop70065-bib-0028] Chen, X. , Wang, Y. , Liu, L. , Cui, J. , Gan, M. , Shum, D. H. K. , & Chan, R. C. K. (2015). The effect of implementation intention on prospective memory: A systematic and meta‐analytic review. Psychiatry Research, 226(1), 14–22. 10.1016/j.psychres.2015.01.011 25639373

[bjop70065-bib-0029] * Conner, M. , & Higgins, A. R. (2010). Long‐term effects of implementation intentions on prevention of smoking uptake among adolescents: A cluster randomized controlled trial. Health Psychology, 29(5), 529–538. 10.1037/a0020317 20836608

[bjop70065-bib-0030] * Conner, M. , West, R. , Ruth, S. E. , Bianca, S. M. , Lawton, R. , Meads, D. , Torgerson, C. , Grogan, S. , Scholtens, K. , Cowap, L. , Armitage, C. J. , Schmitt, L. , & Siddiqi, K. (2019). Effectiveness and cost‐effectiveness of repeated implementation intention formation on adolescent smoking initiation: A cluster randomized controlled trial. Journal of Consulting and Clinical Psychology, 87(5), 422–432. 10.1037/ccp0000387 PMC6474716 30843703

[bjop70065-bib-0031] * Crăciun, C. , & Băban, A. (2008). The role of self‐efficacy, past habits, and action plans in children's eating habits. Cogniţie Creier Comportament, 12(2), 205–218.

[bjop70065-bib-0033] Cunningham, M. R. , & Baumeister, R. F. (2016). How to make nothing out of something: Analyses of the impact of study sampling and statistical interpretation in misleading meta‐analytic conclusions. Frontiers in Psychology, 7, 1639. 10.3389/fpsyg.2016.01639 PMC5079083 27826272

[bjop70065-bib-0034] de Nooijer, J. , Vet, E. , Brug, J. , & de Vries, N. K. (2006). Do implementation intentions help to turn good intentions into higher fruit intakes? Journal of Nutrition Education and Behavior, 38(1), 25–29. 10.1016/j.jneb.2005.11.021 16595275

[bjop70065-bib-0035] de Vet, E. , Oenema, A. , Sheeran, P. , & Brug, J. (2009). Should implementation intentions interventions be implemented in obesity prevention: The impact of if‐then plans on daily physical activity in Dutch adults. International Journal of Behavioral Nutrition and Physical Activity, 6(1), 11. 10.1186/1479-5868-6-11 PMC2662780 19267889

[bjop70065-bib-0036] Diamond, A. (2002). Normal development of prefrontal cortex from birth to young adulthood: Cognitive functions, anatomy, and biochemistry. In D. T. Stuss & R. T. Knight (Eds.), Principles of frontal lobe function (pp. 466–503). Oxford University Press. 10.1093/acprof:oso/9780195134971.003.0029

[bjop70065-bib-0037] Diamond, A. (2013). Executive functions. Annual Review of Psychology, 64(1), 135–168. 10.1146/annurev-psych-113011-143750 PMC4084861 23020641

[bjop70065-bib-0143] * Duckworth, A. L. , Kirby, T. A. , Gollwitzer, A. , & Oettingen, G. (2013). From fantasy to action: Mental contrasting with implementation intentions (MCII) improves academic performance in children. Social Psychological and Personality Science, 4(6), 745–753. 10.1177/1948550613476307 PMC4106484 25068007

[bjop70065-bib-0038] Duckworth, A. L. , Taxer, J. L. , Eskreis‐Winkler, L. , Galla, B. M. , & Gross, J. J. (2019). Self‐control and academic achievement. Annual Review of Psychology, 70(1), 373–399. 10.1146/annurev-psych-010418-103230 30609915

[bjop70065-bib-0039] Dweck, C. S. (1986). Motivational processes affecting learning. American Psychologist, 41(10), 1040–1048. 10.1037/0003-066X.41.10.1040

[bjop70065-bib-0040] Egger, M. , Smith, G. D. , Schneider, M. , & Minder, C. (1997). Bias in meta‐analysis detected by a simple, graphical test. BMJ, 315(7109), 629–634. 10.1136/bmj.315.7109.629 PMC2127453 9310563

[bjop70065-bib-0041] Eldridge, S. , Campbell, M. , Campbell, M. , Drahota‐Towns, A. , Giraudeau, B. , Higgins, J. , & Siegfried, N. (2016). Revised Cochrane risk of bias tool for randomized trials (RoB 2.0) Additional considerations for cluster‐randomized trials . https://sites.google.com/site/riskofbiastool/welcome/rob‐2‐0‐tool

[bjop70065-bib-0042] Fuster, J. M. (2002). Frontal lobe and cognitive development. Journal of Neurocytology, 31(3/5), 373–385. 10.1023/A:1024190429920 12815254

[bjop70065-bib-0043] * Gawrilow, C. , & Gollwitzer, P. M. (2008). Implementation intentions facilitate response inhibition in children with ADHD. Cognitive Therapy and Research, 32(2), 261–280. 10.1007/s10608-007-9150-1

[bjop70065-bib-0044] * Gawrilow, C. , Gollwitzer, P. M. , & Oettingen, G. (2011a). If‐then plans benefit executive functions in children with ADHD. Journal of Social and Clinical Psychology, 30(6), 616–646. 10.1521/jscp.2011.30.6.616

[bjop70065-bib-0045] * Gawrilow, C. , Gollwitzer, P. M. , & Oettingen, G. (2011b). If‐then plans benefit delay of gratification performance in children with and without ADHD. Cognitive Therapy and Research, 35(5), 442–455. 10.1007/s10608-010-9309-z

[bjop70065-bib-0046] * Gawrilow, C. , Morgenroth, K. , Schultz, R. , Oettingen, G. , & Gollwitzer, P. M. (2013). Mental contrasting with implementation intentions enhances self‐regulation of goal pursuit in schoolchildren at risk for ADHD. Motivation and Emotion, 37(1), 134–145. 10.1007/s11031-012-9288-3

[bjop70065-bib-0047] * Gholami, M. , Knoll, N. , & Schwarzer, R. (2015). A brief self‐regulatory intervention increases dental flossing in adolescent girls. International Journal of Behavioral Medicine, 22(5), 645–651. 10.1007/s12529-014-9459-6 25549910

[bjop70065-bib-0048] Gollwitzer, P. M. (1990). Action phases and mind‐sets. In E. T. Higgins & R. M. Sorrentino (Eds.), The handbook of motivation and cognition: Foundations of social behavior (Vol. 2, pp. 53–92). Guilford Press.

[bjop70065-bib-0049] Gollwitzer, P. M. (1999). Implementation intentions: Strong effects of simple plans. American Psychologist, 54(7), 493–503. 10.1037/0003-066X.54.7.493

[bjop70065-bib-0050] Gollwitzer, P. M. (2014). Weakness of the will: Is a quick fix possible? Motivation and Emotion, 38(3), 305–322. 10.1007/s11031-014-9416-3

[bjop70065-bib-0051] Gollwitzer, P. M. , & Brandstätter, V. (1997). Implementation intentions and effective goal pursuit. Journal of Personality and Social Psychology, 73(1), 186–199. 10.1037//0022-3514.81.5.946 11708569

[bjop70065-bib-0052] Gollwitzer, P. M. , & Schaal, B. (1998). Metacognition in action: The importance of implementation intentions. Personality and Social Psychology Review, 2(2), 124–136. 10.1207/s15327957pspr0202_5 15647140

[bjop70065-bib-0053] Gollwitzer, P. M. , & Sheeran, P. (2006). Implementation intentions and goal achievement: A meta‐analysis of effects and processes. Advances in Experimental Social Psychology, 38, 69–119. 10.1016/S0065-2601(06)38002-1

[bjop70065-bib-0054] Griffin, J. W. (2021). Calculating statistical power for meta‐analysis using metapower. The quantitative methods for Psychology, 17(1), 24–39. 10.20982/tqmp.17.1.p024

[bjop70065-bib-0055] Hagger, M. S. , & Luszczynska, A. (2014). Implementation intention and action planning interventions in health contexts: State of the research and proposals for the way forward. Applied Psychology. Health and Well‐Being, 6(1), 1–47. 10.1111/aphw.12017 24591064

[bjop70065-bib-0056] Haith, M. M. (1998). The development of future thinking as essential for the emergence of skill in planning. In S. L. Friedman & E. K. Scholnick (Eds.), The Developmental Psychology of planning: Why, how, and when do we plan? (pp. 25–42). Psychology Press.

[bjop70065-bib-0057] Hall, P. A. , Zehr, C. , Paulitzki, J. , & Rhodes, R. (2014). Implementation intentions for physical activity behavior in older adult women: An examination of executive function as a moderator of treatment effects. Annals of Behavioral Medicine, 48(1), 130–136. 10.1007/s12160-013-9582-7 24500080

[bjop70065-bib-0058] Heckhausen, H. , & Kuhl, J. (1985). From wishes to action: The dead ends and short cuts on the long way to action. In M. Frese & J. Sabini (Eds.), Goal directed behavior: The concept of action in psychology (pp. 134–159). Erlbaum.

[bjop70065-bib-0059] Hedges, L. V. , & Olkin, L. (1985). Statistical methods for meta‐analysis. Academic Press.

[bjop70065-bib-0060] * Higgins, A. , & Conner, M. (2003). Understanding adolescent smoking: The role of the theory of planned behaviour and implementation intentions. Psychology, Health & Medicine, 8(2), 173–186. 10.1080/1354850031000087555

[bjop70065-bib-0061] Higgins, J. P. T. , Thomas, J. , Chandler, J. , Cumpston, M. , Li, T. , Page, M. J. , & Welch, V. A. (2022). Cochrane handbook for systematic reviews of interventions version 6.3 . www.training.cochrane.org/handbook. 10.1002/14651858.ED000142 PMC10284251 31643080

[bjop70065-bib-0062] Janczyk, M. , Dambacher, M. , Bieleke, M. , & Gollwitzer, P. M. (2015). The benefit of no choice: Goal‐directed plans enhance perceptual processing. Psychological Research, 79(2), 206–220. 10.1007/s00426-014-0549-5 24619532

[bjop70065-bib-0063] Kaup, B. , Ulrich, R. , Bausenhart, K. M. , Bryce, D. , Butz, M. V. , Dignath, D. , Dudschig, C. , Franz, V. H. , Friedrich, C. , Gawrilow, C. , Heller, J. , Huff, M. , Hütter, M. , Janczyk, M. , Leuthold, H. , Mallot, H. , Nürk, H.‐C. , Ramscar, M. , Said, N. , … Wong, H. Y. (2024). Modal and amodal cognition: An overarching principle in various domains of psychology. Psychological Research, 88(2), 307–337. 10.1007/s00426-023-01878-w PMC10857976 37847268

[bjop70065-bib-0064] Keller, L. , Gollwitzer, P. M. , & Sheeran, P. (2020). Changing behavior using the model of action phases. In M. S. Hagger , L. D. Cameron , K. Hamilton , N. Hankonen , & T. Lintunen (Eds.), The handbook of behavior change (1st ed., pp. 77–88). Cambridge University Press. 10.1017/9781108677318.006

[bjop70065-bib-0065] Kossmeier, M. , Tran, U. , & Voracek, M. (2020). Metaviz: Forest plots, funnel plots, and visual funnel plot inference for meta‐analysis . R Package Version 0.3.1. https://CRAN.R‐project.org/package=metaviz.

[bjop70065-bib-0066] Kretschmer‐Trendowicz, A. , Kliegel, M. , Goschke, T. , & Altgassen, M. (2021). If‐then’ but when? Effects of implementation intentions on children's and adolescents' prospective memory. Cognitive Development, 57, 100998. 10.1016/j.cogdev.2020.100998

[bjop70065-bib-0067] Kwon, S. C. , Tandon, S. D. , Islam, N. , Riley, L. , & Trinh‐Shevrin, C. (2018). Applying a community‐based participatory research framework to patient and family engagement in the development of patient‐centered outcomes research and practice. Translational Behavioral Medicine, 8(5), 683–691. 10.1093/tbm/ibx026 PMC6128966 30202926

[bjop70065-bib-0068] Latham, G. P. , & Locke, E. A. (1979). Goal setting—A motivational technique that works. Organizational Dynamics, 8(2), 68–80. 10.1016/0090-2616(79)90032-9

[bjop70065-bib-0069] Latimer, A. E. , Ginis, K. A. M. , & Arbour, K. P. (2006). The efficacy of an implementation intention intervention for promoting physical activity among individuals with spinal cord injury: A randomized controlled trial. Rehabilitation Psychology, 51(4), 273–280. 10.1037/0090-5550.51.4.273

[bjop70065-bib-0070] Lengfelder, A. , & Gollwitzer, P. M. (2001). Reflective and reflexive action control in patients with frontal brain lesions. Neuropsychology, 15(1), 80–100. 10.1037/0894-4105.15.1.80 11216892

[bjop70065-bib-0071] Luna, B. , Garver, K. E. , Urban, T. A. , Lazar, N. A. , & Sweeney, J. A. (2004). Maturation of cognitive processes from late childhood to adulthood. Child Development, 75(5), 1357–1372. 10.1111/j.1467-8624.2004.00745.x 15369519

[bjop70065-bib-0072] Luszczynska, A. (2006). An implementation intentions intervention, the use of a planning strategy, and physical activity after myocardial infarction. Social Science & Medicine, 62(4), 900–908. 10.1016/j.socscimed.2005.06.043 16095786

[bjop70065-bib-0073] Malaguti, A. , Ciocanel, O. , Sani, F. , Dillon, J. F. , Eriksen, A. , & Power, K. (2020). Effectiveness of the use of implementation intentions on reduction of substance use: A meta‐analysis. Drug and Alcohol Dependence, 214, 108120. 10.1016/j.drugalcdep.2020.108120 32622228

[bjop70065-bib-0144] Masicampo, E. J. , & Baumeister, R. F. (2012). Committed but closed‐minded: When making a specific plan for a goal hinders success. Social Cognition, 130(1), 37–55. 10.1521/soco.2012.30.1.37

[bjop70065-bib-0074] * Mau, G. , Schramm‐Klein, H. , Schuhen, M. , & Steinmann, S. (2019). What helps children resist distracting marketing stimuli? Implementation intentions and restrictions alter food choice. Appetite, 140, 159–168. 10.1016/j.appet.2019.05.016 31103444

[bjop70065-bib-0075] * Mauduy, M. , Bagneux, V. , & Sénémeaud, C. (2023). Fostering victim‐defending behaviors among school bullying witnesses: A longitudinal and experimental test of two new strategies for changing behavior. Social Psychology of Education, 26(1), 263–274. 10.1007/s11218-022-09745-z

[bjop70065-bib-0076] McClelland, M. , Geldhof, J. , Morrison, F. , Gestsdóttir, S. , Cameron, C. , Bowers, E. , Duckworth, A. , Little, T. , & Grammer, J. (2018). Self‐regulation. In N. Halfon , C. B. Forrest , R. M. Lerner , & E. M. Faustman (Eds.), Handbook of life course health development (pp. 275–298). Springer International Publishing. 10.1007/978-3-319-47143-3_12

[bjop70065-bib-0077] McCrea, S. M. , Mueller, J. H. , & Parrila, R. K. (1999). Quantitative analyses of schooling effects on executive function in young children. Child Neuropsychology, 5(4), 242–250. 10.1076/0929-7049(199912)05:04;1-R;FT242 10925708

[bjop70065-bib-0078] McWilliams, L. , Bellhouse, S. , Yorke, J. , Lloyd, K. , & Armitage, C. J. (2019). Beyond “planning”: A meta‐analysis of implementation intentions to support smoking cessation. Health Psychology, 38(12), 1059–1068. 10.1037/hea0000768 31414843

[bjop70065-bib-0079] * Mischel, W. , & Patterson, C. J. (1976). Substantive and structural elements of effective plans for self‐control. Journal of Personality and Social Psychology, 34(5), 942–950. 10.1037/0022-3514.34.5.942

[bjop70065-bib-0080] Moersdorf, L. , Daum, M. M. , & Freund, A. M. (2022). For whom is the path the goal? A lifespan perspective on the development of goal focus. Collabra: Psychology, 8(1), 31603. 10.1525/collabra.31603

[bjop70065-bib-0081] Moffitt, T. E. , Arseneault, L. , Belsky, D. , Dickson, N. , Hancox, R. J. , Harrington, H. , Houts, R. , Poulton, R. , Roberts, B. W. , Ross, S. , Sears, M. R. , Thomson, W. M. , & Caspi, A. (2011). A gradient of childhood self‐control predicts health, wealth, and public safety. Proceedings of the National Academy of Sciences, 108(7), 2693–2698. 10.1073/pnas.1010076108 PMC3041102 21262822

[bjop70065-bib-0082] Montroy, J. J. , Bowles, R. P. , Skibbe, L. E. , McClelland, M. M. , & Morrison, F. J. (2016). The development of self‐regulation across early childhood. Developmental Psychology, 52(11), 1744–1762. 10.1037/dev0000159 PMC5123795 27709999

[bjop70065-bib-0083] Morrison, F. J. , Ponitz, C. C. , & McClelland, M. M. (2010). Self‐regulation and academic achievement in the transition to school. In S. D. Calkins & M. A. Bell (Eds.), Child development at the intersection of emotion and cognition (pp. 203–224). American Psychological Association. 10.1037/12059-011

[bjop70065-bib-0084] Nagata, J. M. , Memon, Z. , Talebloo, J. , Li, K. , Low, P. , Shao, I. Y. , Ganson, K. T. , Testa, A. , He, J. , Brindis, C. D. , & Baker, F. C. (2025). Prevalence and patterns of social media use in early adolescents. Academic Pediatrics, 25(4), 102784. 10.1016/j.acap.2025.102784 PMC12048217 39800219

[bjop70065-bib-0085] Naumann, A. , Musow, S. , & Katstaller, M. (2020). Instructional sensitivity as a prerequisite for determining the effectiveness of interventions in educational research. In H. Astleitner (Ed.), Intervention research in educational practice: Alternative theoretical frameworks and application problems (pp. 147–170). Waxmann.

[bjop70065-bib-0086] Nosek, B. A. , Ebersole, C. R. , DeHaven, A. C. , & Mellor, D. T. (2018). The preregistration revolution. Proceedings of the National Academy of Sciences, 115(11), 2600–2606. 10.1073/pnas.1708274114 PMC5856500 29531091

[bjop70065-bib-0087] Oettingen, G. , Pak, H. , & Schnetter, K. (2001). Self‐regulation of goal‐setting: Turning free fantasies about the future into binding goals. Journal of Personality and Social Psychology, 80(5), 736–753. 11374746

[bjop70065-bib-0088] Page, M. J. , Moher, D. , Bossuyt, P. M. , Boutron, I. , Hoffmann, T. C. , Mulrow, C. D. , Shamseer, L. , Tetzlaff, J. M. , Akl, E. A. , Brennan, S. E. , Chou, R. , Glanville, J. , Grimshaw, J. M. , Hróbjartsson, A. , Lalu, M. M. , Li, T. , Loder, E. W. , Mayo‐Wilson, E. , McDonald, S. , … McKenzie, J. E. (2021). PRISMA 2020 explanation and elaboration: Updated guidance and exemplars for reporting systematic reviews. BMJ, n160. 10.1136/bmj.n160 PMC8005925 33781993

[bjop70065-bib-0089] Parks‐Stamm, E. J. , Gollwitzer, P. M. , & Oettingen, G. (2007). Action control by implementation intentions: Effective cue detection and efficient response initiation. Social Cognition, 25(2), 248–266. 10.1521/soco.2007.25.2.248

[bjop70065-bib-0090] * Patterson, C. J. , & Mischel, W. (1975). Plans to resist distraction. Developmental Psychology, 11(3), 369–378. 10.1037/h0076565

[bjop70065-bib-0091] * Patterson, C. J. , & Mischel, W. (1976). Effects of temptation‐inhibiting and task‐facilitating plans on self‐control. Journal of Personality and Social Psychology, 33(2), 209–217. 10.1037/0022-3514.33.2.209

[bjop70065-bib-0092] * Paul, I. , Gawrilow, C. , Zech, F. , Gollwitzer, P. , Rockstroh, B. , Odenthal, G. , Kratzer, W. , & Wienbruch, C. (2007). If–then planning modulates the P300 in children with attention deficit hyperactivity disorder. Neuroreport, 18(7), 653–657. 10.1097/WNR.0b013e3280bef966 17426593

[bjop70065-bib-0093] Perry, N. B. , Calkins, S. D. , Dollar, J. M. , Keane, S. P. , & Shanahan, L. (2018). Self‐regulation as a predictor of patterns of change in externalizing behaviors from infancy to adolescence. Development and Psychopathology, 30(2), 497–510. 10.1017/S0954579417000992 PMC5858969 28641597

[bjop70065-bib-0094] Podell, J. L. , Mychailyszyn, M. , Edmunds, J. , Puleo, C. M. , & Kendall, P. C. (2010). The coping cat program for anxious youth: The FEAR plan comes to life. Cognitive and Behavioral Practice, 17(2), 132–141. 10.1016/j.cbpra.2009.11.001 PMC2950324 20936081

[bjop70065-bib-0095] Posit team . (2025). RStudio: Integrated development environment for R. Posit Software, PBC. https://www.posit.co/

[bjop70065-bib-0096] Prestwich, A. , Perugini, M. , & Hurling, R. (2009). Can the effects of implementation intentions on exercise be enhanced using text messages? Psychology and Health, 24(6), 677–687. 10.1080/08870440802040715 20205020

[bjop70065-bib-0097] Prestwich, A. , Perugini, M. , & Hurling, R. (2010). Can implementation intentions and text messages promote brisk walking? A Randomized Trial. Health Psychology, 29(1), 40–49. 10.1037/a0016993 20063934

[bjop70065-bib-0098] Pustejovsky, J. E. , & Rodgers, M. A. (2019). Testing for funnel plot asymmetry of standardized mean differences. Research Synthesis Methods, 10(1), 57–71. 10.1002/jrsm.1332 30506832

[bjop70065-bib-0099] R Core Team . (2024). R: A language and environment for statistical computing. R Foundation for Statistical Computing. https://www.r‐project.org/

[bjop70065-bib-0100] Rhodes, R. E. , Grant, S. , & De Bruijn, G.‐J. (2020). Planning and implementation intention interventions. In M. S. Hagger , L. D. Cameron , K. Hamilton , N. Hankonen , & T. Lintunen (Eds.), The handbook of behavior change (1st ed., pp. 572–585). Cambridge University Press. 10.1017/9781108677318.039

[bjop70065-bib-0101] Rodrigues, R. C. M. , São João, T. M. , Gallani, M. C. B. J. , Cornélio, M. E. , & Alexandre, N. M. C. (2013). The “moving heart program”: An intervention to improve physical activity among patients with coronary heart disease. Revista Latino‐Americana de Enfermagem, 21, 180–189. 10.1590/s0104-11692013000700023 23459906

[bjop70065-bib-0102] * Rozendaal, E. , & Figner, B. (2020). Effectiveness of a school‐based intervention to empower children to cope with advertising. Journal of Media Psychology, 32(3), 107–118. 10.1027/1864-1105/a000262

[bjop70065-bib-0104] * Schaaf, M. , Brod, G. , & Breitwieser, J. (2025). Planning promotes studying – The higher the plan quality the better: A micro‐randomized trial. Contemporary Educational Psychology, 83, 102422. 10.1016/j.cedpsych.2025.102422

[bjop70065-bib-0105] * Schneikert, M. (2005). *Der Einfluss von Vorsätzen auf die kognitive Flexibilität von Kindern mit Aufmerksamkeitsdefizit‐/Hyperaktivitätsstörung (ADHS) [The influence of implementation intentions on the cognitive flexibility of children with attention deficit/hyperactivity disorder (ADHD)]*. Unpublished Diploma Thesis, University of Konstanz, Germany.

[bjop70065-bib-0106] * Schunk, D. , Berger, E. M. , Hermes, H. , Winkel, K. , & Fehr, E. (2022). Teaching self‐regulation. Nature Human Behaviour, 6(12), 1680–1690. 10.1038/s41562-022-01449-w 36229604

[bjop70065-bib-0107] * Schwantje, W. (2005). *Blockierung von externen interferierenden Reizen durch Vorsätze: Eine Vergleichsstudie von ADHS‐ und Kontrollkindern [Blocking external interfering stimuli through implementation intentions: A comparative study of children with ADHD and control children]*. Unpublished Diploma Thesis, University of Konstanz, Germany.

[bjop70065-bib-0108] * Schwarz, U. , & Gawrilow, C. (2019). Measuring and compensating for deficits of self‐regulation in school children via ambulatory assessment. Psychology in Russia: State of the Art, 12(4), 8–22. 10.11621/pir.2019.0401

[bjop70065-bib-0109] Sheeran, P. (2002). Intention—Behavior relations: A conceptual and empirical review. European Review of Social Psychology, 12(1), 1–36. 10.1080/14792772143000003

[bjop70065-bib-0110] Sheeran, P. , Listrom, O. , & Gollwitzer, P. M. (2024). The when and how of planning: Meta‐analysis of the scope and components of implementation intentions in 642 tests. European Review of Social Psychology, 36(1), 2334563. 10.1080/10463283.2024.2334563

[bjop70065-bib-0146] Sheeran, P. , Webb, T. L. , & Gollwitzer, P. M. (2005). The interplay between goal intentions and implementation intentions. Personality and Social Psychology Bulletin, 31(1), 87–98. 10.1177/0146167204271308 15574664

[bjop70065-bib-0111] Silva, M. A. V. D. , São‐João, T. M. , Brizon, V. C. , Franco, D. H. , & Mialhe, F. L. (2018). Impact of implementation intentions on physical activity practice in adults: A systematic review and meta‐analysis of randomized clinical trials. PLoS One, 13(11), e0206294. 10.1371/journal.pone.0206294 PMC6235272 30427874

[bjop70065-bib-0112] Simonsohn, U. , Nelson, L. D. , & Simmons, J. P. (2014a). P‐curve and effect size: Correcting for publication bias using only significant results. Perspectives on Psychological Science, 9(6), 666–681. 10.1177/1745691614553988 26186117

[bjop70065-bib-0113] Simonsohn, U. , Nelson, L. D. , & Simmons, J. P. (2014b). P‐curve: A key to the file‐drawer. Journal of Experimental Psychology: General, 143(2), 534–547. 10.1037/a0033242 23855496

[bjop70065-bib-0114] Simonsohn, U. , Simmons, J. P. , & Nelson, L. D. (2015). Better p‐curves: Making p‐curve analysis more robust to errors, fraud, and ambitious p‐hacking, a reply to Ulrich and Miller. Journal of Experimental Psychology: General, 144(6), 1146–1152. 10.1037/xge0000104 26595842

[bjop70065-bib-0115] * Simpriano, D. C. A. B. , & Mialhe, F. L. (2017). Impact of educational interventions based on the implementation intentions strategy on the oral health of schoolchildren. Pesquisa Brasileira em Odontopediatria e Clínica Integrada, 17(1). 10.4034/PBOCI.2017.171.01

[bjop70065-bib-0116] Smith, S. R. , Hagger, M. S. , Keech, J. J. , Moyers, S. A. , & Hamilton, K. (2022). Improving hand hygiene behavior using a novel theory‐based intervention during the COVID‐19 pandemic. Annals of Behavioral Medicine, 56(11), 1157–1173. 10.1093/abm/kaac041 PMC9635998 36099420

[bjop70065-bib-0117] Sniehotta, F. F. (2009). Towards a theory of intentional behaviour change: Plans, planning, and self‐regulation. British Journal of Health Psychology, 14(2), 261–273. 10.1348/135910708X389042 19102817

[bjop70065-bib-0118] Stanley, T. D. (2007). Meta‐regression methods for detecting and estimating empirical effects in the presence of publication selection. Oxford Bulletin of Economics and Statistics, 70, 102–127. 10.1111/j.1468-0084.2007.00487.x

[bjop70065-bib-0119] Stanley, T. D. , & Doucouliagos, H. (2014). Meta‐regression approximations to reduce publication selection bias. Research Synthesis Methods, 5(1), 60–78. 10.1002/jrsm.1095 26054026

[bjop70065-bib-0120] Steinberg, L. (2008). A social neuroscience perspective on adolescent risk‐taking. Developmental Review, 28(1), 78–106. 10.1016/j.dr.2007.08.002 PMC2396566 18509515

[bjop70065-bib-0121] Steinberg, L. , & Morris, A. S. (2001). Adolescent development. Annual Review of Psychology, 52(1), 83–110. 10.1146/annurev.psych.52.1.83 11148300

[bjop70065-bib-0122] Sterne, J. A. , Hernán, M. A. , Reeves, B. C. , Savović, J. , Berkman, N. D. , Viswanathan, M. , Henry, D. , Altman, D. G. , Ansari, M. T. , Boutron, I. , Carpenter, J. R. , Chan, A.‐W. , Churchill, R. , Deeks, J. J. , Hróbjartsson, A. , Kirkham, J. , Jüni, P. , Loke, Y. K. , Pigott, T. D. , … Higgins, J. P. (2016). ROBINS‐I: A tool for assessing risk of bias in non‐randomised studies of interventions. BMJ, i4919. 10.1136/bmj.i4919 PMC5062054 27733354

[bjop70065-bib-0123] Sterne, J. A. C. , Savović, J. , Page, M. J. , Elbers, R. G. , Blencowe, N. S. , Boutron, I. , Cates, C. J. , Cheng, H.‐Y. , Corbett, M. S. , Eldridge, S. M. , Emberson, J. R. , Hernán, M. A. , Hopewell, S. , Hróbjartsson, A. , Junqueira, D. R. , Jüni, P. , Kirkham, J. J. , Lasserson, T. , Li, T. , … Higgins, J. P. T. (2019). RoB 2: A revised tool for assessing risk of bias in randomised trials. BMJ, l4898. 10.1136/bmj.l4898 31462531

[bjop70065-bib-0124] * Stumpf, N. (2004). Der Einfluss von Vorsätzen auf die Multitaskingfähigkeit von Kindern mit und ohne Aufmerksamkeitsdefizit‐/Hyperaktivitätsstörung (ADHS) [The influence of implementation intentions on the multitasking ability of children with and without attention deficit/hyperactivity disorder (ADHD)]. Unpublished Diploma Thesis. University of Konstanz.

[bjop70065-bib-0125] Thandi, C. S. , Constantinou, S. , Vincent, R. , & Ridd, M. J. (2023). Where and how have written action plans for atopic eczema/dermatitis been developed and evaluated? Systematic review. Skin Health and Disease, 3(3), ski2.213. 10.1002/ski2.213 PMC10233085 37275422

[bjop70065-bib-0126] * Thompson, D. , Bhatt, R. , Vazquez, I. , Cullen, K. W. , Baranowski, J. , Baranowski, T. , & Liu, Y. (2015). Creating action plans in a serious video game increases and maintains child fruit‐vegetable intake: A randomized controlled trial. International Journal of Behavioral Nutrition and Physical Activity, 12(1), 1–10. 10.1186/s12966-015-0199-z PMC4372224 25890060

[bjop70065-bib-0127] Thompson, S. G. , & Sharp, S. J. (1999). Explaining heterogeneity in meta‐analysis: A comparison of methods. Statistics in Medicine, 18(20), 2693–2708. 10.1002/(SICI)1097-0258(19991030)18:20<2693::AID-SIM235>3.0.CO;2-V 10521860

[bjop70065-bib-0128] Toli, A. , Webb, T. L. , & Hardy, G. E. (2016). Does forming implementation intentions help people with mental health problems to achieve goals? A meta‐analysis of experimental studies with clinical and analogue samples. British Journal of Clinical Psychology, 55(1), 69–90. 10.1111/bjc.12086 25965276

[bjop70065-bib-0129] van Aert, R. C. M. , & van Assen, M. A. L. M. (2021). Correcting for publication bias in a meta‐analysis with the p‐uniform* method *OSF preprint*. https://osf.io/ebq6m/. 10.3758/s13423-025-02812-4 PMC12948804 41760983

[bjop70065-bib-0130] van Lissa, C. J. (2020). Small sample meta‐analyses. In I. R. van de Schoot & M. Miočević (Eds.), Small sample size solutions (1st ed., pp. 186–202). Routledge. 10.4324/9780429273872-16

[bjop70065-bib-0131] Viechtbauer, W. , & Cheung, M. W.‐L. (2010). Outlier and influence diagnostics for meta‐analysis. Research Synthesis Methods, 1(2), 112–125. 10.1002/jrsm.11 26061377

[bjop70065-bib-0132] Webb, T. L. , Schweiger Gallo, I. , Miles, E. , Gollwitzer, P. M. , & Sheeran, P. (2012). Effective regulation of affect: An action control perspective on emotion regulation. European Review of Social Psychology, 23(1), 143–186. 10.1080/10463283.2012.718134

[bjop70065-bib-0133] Webb, T. L. , & Sheeran, P. (2008). Mechanisms of implementation intention effects: The role of goal intentions, self‐efficacy, and accessibility of plan components. British Journal of Social Psychology, 47(3), 373–395. 10.1348/014466607X267010 18096108

[bjop70065-bib-0134] Wickham, H. (2016). ggplot2: Elegant graphics for data analysis. Springer‐Verlag.

[bjop70065-bib-0135] Wieber, F. , Odenthal, G. , & Gollwitzer, P. (2010). Self‐efficacy feelings moderate implementation intention effects. Self and Identity, 9(2), 177–194. 10.1080/15298860902860333

[bjop70065-bib-0136] Wieber, F. , & Sassenberg, K. (2006). I can't take my eyes off of it–attention attraction effects of implementation intentions. Social Cognition, 24(6), 723–752. 10.1521/soco.2006.24.6.723

[bjop70065-bib-0137] * Wieber, F. , von Suchodoletz, A. , Heikamp, T. , Trommsdorff, G. , & Gollwitzer, P. M. (2011). If‐then planning helps school‐aged children to ignore attractive distractions. Social Psychology, 42(1), 39–47. 10.1027/1864-9335/a000041

[bjop70065-bib-0138] Wigfield, A. , Klauda, S. L. , & Cambria, J. (2011). Influences on the development of academic self‐regulatory processes. In D. H. Schunk & B. Zimmerman (Eds.), Handbook of self‐regulation of learning and performance (pp. 33–48). Routledge. 10.4324/9780203839010.ch3

[bjop70065-bib-0145] Wittmann, W. W. , & Klumb, P. L. (2006). How to Fool Yourself With Experiments in Testing Theories in Psychological Research. In R. R. Bootzin & P. E. McKnight (Eds.), Strengthening research methodology: Psychological measurement and evaluation (pp. 185–211). American Psychological Association. 10.1037/11384-010

[bjop70065-bib-0140] * Yang, T. X. , Zhang, S. Y. , Wang, Y. , Su, X. M. , Yuan, C. W. , Lui, S. S. Y. , & Chan, R. C. K. (2023). The effect of implementation intentions on event‐, time‐, and activity‐based prospective memory in typically developing children. International Journal of Behavioral Development, 47(2), 146–157. 10.1177/01650254221146420

[bjop70065-bib-0141] Zemek, R. L. , Bhogal, S. K. , & Ducharme, F. M. (2008). Systematic review of randomized controlled trials examining written action plans in children: What is the plan? Archives of Pediatrics & Adolescent Medicine, 162(2), 157–163. 10.1001/archpediatrics.2007.34 18250241

[bjop70065-bib-0142] * Zimmermann, T. D. , & Meier, B. (2010). The effect of implementation intentions on prospective memory performance across the lifespan. Applied Cognitive Psychology, 24(5), 645–658. 10.1002/acp.1576

